# Iodine-125 brachytherapy triggers immunogenic cell death and potentiates anti-PD-L1 immunotherapy in bone metastatic triple-negative breast cancer

**DOI:** 10.3389/fimmu.2026.1761538

**Published:** 2026-03-31

**Authors:** Min Li, Zhenzhen Cui, Jiaxin Yang, Qiyu Sun, Yanbo Hu, Juan Shen, Zhihao Li, Xiaoyang Han, Yixuan Wang, Jiageng Tang, Hengsong Yu, Xiaowen Ma, Jing Wang

**Affiliations:** 1 Department of Nuclear Medicine, Xijing Hospital, Fourth Military Medical University, Xi’an, Shanxi, China; 2 Department of Nuclear Medicine, The 960th Hospital of the People's Liberation Army (PLA), Jinan, Shandong, China; 3 Jinzhou Medical University Graduate Training Base (The 960th Hospital of the PLA), Jinan, Shandong, China; 4 Shandong University of Traditional Chinese Medicine Graduate Training Base (The 960th Hospital of the People's Liberation Army (PLA)), Jinan, Shandong, China; 5 Shandong Second Medical University Graduate Training Base (The 960th Hospital of the People's Liberation Army (PLA)), Jinan, Shandong, China; 6 Department of Pharmacy, The 960th Hospital of the People's Liberation Army (PLA), Jinan, Shandong, China

**Keywords:** bone metastasis, brachytherapy, calreticulin, external beam radiotherapy, immunogenic cell death, iodine-125 seeds, triple-negative breast cancer

## Abstract

**Introduction:**

Triple-negative breast cancer (TNBC) with bone metastasis is challenging to treat due to an immunosuppressive microenvironment and the limited, transient immune activation from conventional external beam radiotherapy. Inducing immunogenic cell death (ICD) offers a strategy to remodel this environment, but its systemic effects remain to be validated. Here, we investigate iodine-125 (^125^I) brachytherapy as a method to induce sustained ICD and activate systemic antitumor immunity in TNBC bone metastasis using transcriptomic analysis, a dual-tumor model, and CALR-targeted molecular imaging.

**Methods:**

A dual-tumor model of 4T1 mouse breast cancer with tibial bone metastasis and distal subcutaneous implantation was established. The therapeutic efficacy of ^¹²⁵^I seeds at different doses (0.3-0.8 mCi), alone or in combination with anti–PD-L1 antibody, was systematically assessed. ICD biomarkers (CALR, HMGB1) and immune cell infiltration were analyzed using Western blot, qRT-PCR, immunohistochemistry, and flow cytometry. Transcriptome sequencing was performed to explore changes in ICD and related immune pathways. In vivo dynamic imaging of ICD was achieved using a CALR-targeting bimodal nanoprobe. Comparative analyses were conducted between ^125^I brachytherapy and EBRT to assess differences in tumor control and immune activation. Treatment sequencing studies were performed to evaluate the optimal combination strategy.

**Results:**

This study confirmed that ^125^I seeds effectively induce sustained immunogenic cell death, significantly upregulating damage-associated molecular patterns (DAMPs) such as CALR and HMGB1. In the 4T1 bone metastasis model, local implantation of ^125^I (0.3-0.8 mCi) inhibited tumor growth in a dose-dependent manner, with the 0.8 mCi group showing the best therapeutic effect and no significant toxicity. Mechanistically, ^125^I promoted CD8α⁺T cell infiltration, activated ICD-related pathways, and triggered systemic antitumor immunity, as demonstrated by inhibition of distant tumor growth in the bilateral tumor system. Compared with EBRT, 125I brachytherapy achieved superior local control and more robust immune activation. Using a CALR-targeting nanoprobe, dynamic *in vivo* imaging of ICD was successfully achieved. Treatment sequencing studies revealed that initiating with ^125^I brachytherapy followed by combination therapy with PD-L1 antibody and Abraxane resulted in the most favorable outcome, confirming this “^125^I-first” approach as an effective immune priming strategy.

**Discussion:**

This study demonstrates that ^125^I brachytherapy overcomes the limitations of conventional radiotherapy by inducing sustained immunogenic cell death, remodeling the tumor immune microenvironment, and activating systemic antitumor immunity. ^125^I seeds not only inhibit the growth of TNBC bone metastases and distal tumors but also significantly enhance the infiltration of CD8α^⁺^T cells into tumor sites. Notably, the sequential regimen of ^125^I brachytherapy followed by PD-L1 antibody and Abraxane shows significant synergistic effects. These findings establish ^125^I brachytherapy as a superior immune-priming modality and provide a mechanistic rationale for integrating low-dose-rate brachytherapy with immunotherapy to overcome resistance in bone-metastatic TNBC, offering strong potential for clinical translation., iodine-125 seeds, triple-negative breast cancer.

## 1 Introduction

Bone is a predominant site of metastasis in triple negative breast cancer (TNBC),occurring in 30–50%of patients and drastically shortening median overall survival (1, 2).While bone targeted agents alleviate skeletal complications, they do not control tumor growth, underscoring the need for effective systemic therapies (3).Immune checkpoint inhibitors (ICIs) benefit some patients with metastatic TNBC (4),yet their efficacy in bone metastases remains strikingly low, with response rates below 10%,far lower than in visceral sites (5).This resistance specific to bone metastasis identifies it as an independent adverse prognostic factor (6, 7),pointing to unique immunobiological barriers within the bone microenvironment.

Bone lesions exhibit an immune cold phenotype, characterized by minimal CD8^+^T cell infiltration and an abundance of immunosuppressive cells (8, 9).Tumor induced osteolysis releases TGF beta, which both promotes tumor progression and broadly suppresses T cell function (10).Furthermore, the hypoxic, acidic, and calcium rich bone milieu impairs antigen presentation and T cell activity (11, 12),creating an immune excluded state. Together, these convergent mechanisms significantly blunt the activity of PD-1/PD-L1 blockade in patients with bone metastatic TNBC ( (13).

Radiotherapy is a cornerstone of palliative care for bone metastases and can, in principle, reverse immunosuppression by inducing immunogenic cell death (ICD) (9, 14).However, converting radiation induced ICD into productive immunity requires a sustained danger signal, a requirement that is fundamentally mismatched to the biological and physical characteristics of conventional external beam radiotherapy (EBRT).The brief, high dose pulses of EBRT generate only transient DAMP release, a signal that is rapidly extinguished in the hypoxic bone niche where ROS generation and ICD are dampened (15, 16).Concurrent upregulation of HIF-1α and TGF-β further recruits immunosuppressive cells (17, 18).Consequently, even dose escalated or altered fractionation schedules fail to achieve durable immune activation (19, 20),which fundamentally restricts the potential of EBRT to act as an effective immune primer within the immunosuppressive bone metastatic microenvironment.

In this context, low-dose-rate brachytherapy(LDR) using iodine-125(^125^I) seeds emerges as a strategically distinct approach. By delivering continuous, low level irradiation over several weeks,^125^I seeds transform the temporal paradigm of radiotherapy. This protracted exposure aligns with the biological timeline required for effective immune synapse formation and clonal expansion. Mechanistically, it promotes sustained tumor reoxygenation, prolonged endoplasmic reticulum stress, and the gradual, cumulative release of damage associated molecular patterns, thereby converting the tumor into a persistent *in situ* vaccine factory (21–23).Retrospective clinical observations support this potential, indicating that ^125^I implantation may offer superior local control and a higher propensity to elicit abscopal effects compared with EBRT (24, 25).

This question underscores three critical knowledge gaps that hinder the rational integration of radiotherapy and immunotherapy for bone metastatic TNBC. First, direct comparisons of the potency and persistence of ICD induced by LDR versus EBRT are scarce. Second, tools to visualize ICD dynamics *in vivo* are lacking, which impedes treatment optimization. Third, the optimal strategy for sequencing LDR brachytherapy with ICIs remains undefined.

To address these gaps, we conducted a comprehensive study to decode the immunogenic properties of ^125^I brachytherapy. Our aims were to:(i) directly compare the temporal dynamics of ICD induced by ^125^I and EBRT;(ii) develop a calreticulin targeted molecular probe for real time visualization of ICD;(iii) quantify systemic immunity and abscopal effects using bilateral tumor models and (iv) establish an optimized ^125^I first sequencing strategy with anti PD-L1 therapy. This work elucidates how sustained LDR irradiation overcomes bone specific immunosuppression and provides a translatable paradigm for combination radio immunotherapy.

## 2 Materials and methods

### 2.1 Cell lines and reagents

Murine breast cancer 4T1 cells and human umbilical vein endothelial cells (HUVECs) were obtained from ATCC. 4T1 cells were cultured in RPMI-1640 supplemented with 10% FBS and 1% penicillin–streptomycin, while HUVECs were maintained in EGM-2 medium containing 5% FBS and 1% penicillin–streptomycin. All cells were incubated at 37 °C in a humidified 5% CO_2_ atmosphere and passaged at 80–90% confluence.

Radioactive ^125^I seeds were purchased from North China Pharmaceutical Group Corporation.

### 2.2 Cell proliferation assay

4T1 and HUVEC cells in the logarithmic phase were seeded into 96-well plates at a density of 1×10^4^ cells per well in 100 µL of complete medium and incubated overnight. The medium was then replaced with serum-free RPMI-1640 containing varying concentrations of ^125^I seeds, followed by incubation for 24 or 48 h. Subsequently,10 µL of CCK-8 reagent(Vazyme, Nanjing, China) was added to each well and incubated for 2 h at 37 °C. Absorbance was measured at 450 nm using a microplate reader (BioTek, USA).The inhibition rate was calculated as follows: Inhibition rate (%)=[1−(A_treatment_/A_control_)]×100%.

### 2.3 TUNEL assay for apoptosis detection

Apoptosis was evaluated using a TUNEL Apoptosis Detection Kit(#C1089, Beyotime, Shanghai, China) according to the manufacturer’s instructions. 4T1 cells were seeded in 6-well plates at 1×10^6^ cells per well and incubated overnight. Cells were then exposed to indicated doses of ^125^I for 12 h. After treatment, cells were fixed with 4% paraformaldehyde for 30 min at room temperature and incubated with TUNEL working solution at 37 °C for 1 h in the dark.

Fluorescence images were captured using an Olympus fluorescence microscope(Japan).TUNEL-positive apoptotic cells were visualized as red fluorescence under Cy3 excitation/emission (Ex=550 nm, Em=570 nm).

### 2.4 Establishment of a Murine TNBC bone metastatic model

Female BALB/c mice (3-4 weeks old) were purchased from Xingkang Laboratory Animal Center (Jinan, China) and housed under specific pathogen-free (SPF) conditions at 25 ± 2°C and 40–50% relative humidity, with free access to food and water. Prior to tumor implantation, the mice were acclimated for one week in a barrier facility. All experimental procedures were approved by the Ethics Committee of the 960th Hospital of the Joint Logistics Support Force and were conducted in strict accordance with relevant regulations (Ethics Review No. 184, 2025).

After the acclimation period, 4T1 cells (1×10^6^) suspended in sterile RPMI-1640 medium were injected into the tibial marrow cavity to establish a TNBC bone metastasis model. When tumors reached approximately 100mm^3^, mice were randomly assigned to five groups (n=9 per group): control, ¹²^5^I-0.3mCi, ¹²^5^I-0.6mCi, ¹²^5^I-0.8mCi, and external beam radiotherapy (EBRT, three fractions of 8 Gy). At 7, 14, and 21 days post-treatment, three mice from each group were euthanized for analysis. Body weight and tumor volume were recorded daily. Tumor volume (TV) was calculated using the formula: TV=(Length × Width²)/2.

### 2.5 Hematoxylin and eosin staining

Upon completion of the experiment, tumors and major organs were harvested and fixed in 4% paraformaldehyde for 24 h. Fixed tissues were processed, paraffin-embedded, and sectioned at 5-8 µm thickness. Sections were baked at 60 °C for 2 h, deparaffinized in xylene, and rehydrated through graded ethanol.

The rehydrated sections were stained with hematoxylin (#G1004, Servicebio, China) and eosin (#G1001, Servicebio, China), rinsed with distilled water, and mounted with neutral resin. Histopathological features were observed under a light microscope (Olympus, Japan).

### 2.6 Immunohistochemistry

Paraffin-embedded tumor sections were deparaffinized, rehydrated through graded ethanol, and subjected to antigen retrieval in sodium citrate buffer (pH 6.0) at 90 °C for 10 min. After cooling to room temperature, sections were washed with phosphate-buffered saline (PBS), permeabilized with 0.5% Triton X-100 for 20 min, and treated with 3% H_2_O_2_ in methanol for 10 min to block endogenous peroxidase activity. Non-specific binding was blocked with 5% bovine serum albumin (BSA) for 1 h at room temperature.

Sections were incubated overnight at 4 °C with primary antibodies against HMGB1 (1:500, #10829-1-AP, Proteintech), CALR (1:1000, #10292-1-AP, Proteintech), CD4(1:200,#25229,Cell Signaling Technology)and CD8 (1:200, #85336S, Cell Signaling Technology), CD86 (1:200,#91882, Cell Signaling Technology), 8-OHdG (1:200, #ab48508, Abcam), 4-HNE (1:200, #ab46545, Abcam). After PBS washing, slides were incubated with HRP-conjugated secondary antibodies for 1 h at room temperature. Signals were visualized using a diaminobenzidine (DAB) substrate kit, and the reaction was terminated by rinsing with distilled water. Sections were counterstained with hematoxylin, dehydrated, cleared, and mounted with neutral resin. Images were acquired under a bright-field microscope (Olympus, Japan).

### 2.7 RNA extraction and quantitative PCR analysis

Total RNA was extracted from cells and tumor tissues using a commercial RNA extraction kit. Complementary DNA (cDNA) was synthesized from 1 µg of total RNA using a reverse transcription kit (#9108, Takara, Japan) according to the manufacturer’s instructions. Quantitative real-time PCR (qPCR) was performed to assess HMGB1 and CALR expression. Gene-specific primers (sequences listed in **Supplementary Table 1**) were designed based on NCBI reference sequences and synthesized by I-Tongyong Bio (Anhui, China). Relative gene expression was calculated using the 2^^–ΔΔCT^ method and normalized to GAPDH.

### 2.8 Western blot analysis

Cells or tumor tissues were lysed on ice using RIPA buffer (P0013B, Beyotime). Lysates were mixed with 5×loading buffer to prepare 1×samples, heated at 100 °C for 10 min, and loaded onto SDS-PAGE gels for electrophoretic separation. Proteins were transferred to PVDF membranes (Millipore), blocked with 5% BSA for 1 h at room temperature, and incubated overnight at 4°C with primary antibodies against HMGB1 (1:1000, #6893, CST), CALR (1:1000, #12238, CST), Flotillin-2 (1:1000, #3436, CST), and β-actin (1:2000, #10624-2-AP, Proteintech). Membranes were then incubated with HRP-conjugated secondary antibody (goat anti-rabbit IgG, #A21020, Abbkine) for 1 h at room temperature. Protein bands were visualized using an ECL substrate (#P1008M, Beyotime) and imaged with a Tanon 5200 chemiluminescence system. Band intensities were quantified using Image Lab software (BIO-RAD).

### 2.9 Enzyme-linked immunosorbent assay for HMGB1

The concentration of HMGB1 in 4T1 cell culture supernatants and mouse serum was measured using a commercial ELISA kit (#12907, Jiangsu Meian Biotechnology, China) according to the manufacturer’s instructions. For *in vitro* experiments, 4T1 cells were treated with different doses of ¹²^5^I, and supernatants were collected at specified time points, centrifuged at 12,000×g for 10 min at 4 °C, filtered through a 0.22 µm membrane, and stored at -80 °C until analysis. For *in vivo* experiments, blood samples were obtained from the orbital sinus on days 7, 14, and 21 post-treatment. After clotting at room temperature for 30 min, serum was separated by centrifugation at 2,000×g for 10 min and stored at -80 °C. Absorbance was measured at 450 nm using a microplate reader.

### 2.10 PET/CT image acquisition and analysis

PET/CT imaging was performed one week after establishment of the 4T1 bone metastasis model (pre-treatment) and on days 7 and 14 post-treatment. Mice were intravenously injected with ^18^F-FDG (0.1 mCi, ~3.7 MBq) and allowed to rest for 30-60 min to enable tracer uptake. PET scans were acquired for 10-15 min to evaluate ^18^F-FDG accumulation in tumors, and metabolic activity was quantified using the standardized uptake value (SUV). CT scans were subsequently performed to localize tumors, assess size and position, and evaluate bone metastasis. PET and CT images were co-registered using fusion software for integrated analysis.

### 2.11 Dual-tumor mouse model

1×10^6^ 4T1 breast cancer cells suspended in sterile RPMI-1640 medium were injected into the tibial plateau to induce bone metastases. When the bone tumors reached 100 mm^3^, mice were randomly assigned to a control group or ^125^I treatment groups (0.3, 0.6, or 0.8 mCi; n=5) and received local implantation of ¹²^5^I seeds for 14 days. Fourteen days after seed implantation, a secondary distal tumor was established by subcutaneous injection of 5×10^5^ 4T1 cells into the dorsal flank.

### 2.12 Synthesis and characterization of the CALR-targeted probes

The CALR-targeted fluorescent probes FITC-KLGFFKR and KLGFFKRC-Mal-Cy5.5 were synthesized commercially (Xi’an Ruixi Biological Technology Co., Ltd., China) and rigorously characterized to validate their biochemical specificity.

### 2.13 Confocal microscopy for probe localization

4T1 and HUVECs cells were seeded in confocal dishes and exposed to 9.25×10^3^ kBq/mL ^125^I for 4 h. Cells were then incubated with KLGFFKR-FITC nanoprobes (5 μg/mL) for 2 h, washed, fixed with 4% paraformaldehyde, and blocked with 5% BSA or goat serum. Nuclei were counterstained with DAPI (1 μg/mL), and nanoprobe localization was visualized using a confocal laser scanning microscope.

### 2.14 *In vivo* fluorescence imaging for nanoprobe targeting

In the 4T1 bone metastasis model, mice were randomly assigned to control or ^125^I treatment groups (0.8 mCi/mouse; n=3) at 7 or 14 days post-tumor inoculation. Mice received 100 μL of KLGFFKR-Mal-Cy5.5 probes via tail vein injection. One hour after administration, *in vivo* fluorescence imaging was performed using the IVIS Lumina XR system.

### 2.15 Transcriptome sequencing and pathway enrichment analysis

Tumor tissues were harvested from the model control and ¹²^5^I-treated groups on days 7, 14, and 21 post-treatment for RNA sequencing (RNA-seq). Raw read counts were normalized and variance-stabilized using the rlog (regularized log) transformation in the DESeq2 package. Gene Set Enrichment Analysis (GSEA) was performed using the Hallmark gene sets (MSigDB v7.4) to identify significantly enriched pathways. The signal-to-noise ratio was used for gene ranking, and significance was assessed through 10,000 permutations to ensure statistical robustness.

### 2.16 Analysis of ICD−related gene expression

The expression matrix was variance-stabilized using the rlog (regularized log) transformation in DESeq2.Representative genes associated with ICD were selected, and their expression levels were compared between the model control and treatment groups at different time points.

### 2.17 Mouse model and pharmacological modulation of ROS signaling

Female BALB/c mice (6-8 weeks old) were used to establish a 4T1 bone metastasis model by intratibial injection of 4T1 murine triple-negative breast cancer cells, as described above. Tumor-bearing mice were randomly assigned to four groups: model (untreated control), ^125^I brachytherapy (0.8 mCi), ^125^I plus N-acetylcysteine (NAC), and ^125^I plus MitoQ. For brachytherapy, a single 0.8 mCi ^125^I seed was implanted locally into the tumor-bearing tibia under sterile conditions once tumors were established.

To pharmacologically modulate ROS signaling, NAC, a pan-ROS scavenger, or MitoQ, a mitochondria-targeted antioxidant, was administered intraperitoneally beginning one day prior to ^125^I implantation and continued daily until the experimental endpoint. NAC was given at 150 mg/kg/day, and MitoQ at 10 mg/kg/day; mice in the model and ^125^I-only groups received equivalent volumes of vehicle. Tumor size was measured daily using digital calipers, and tumor volume was calculated as (Length×Width^2^)/2. Body weight was monitored throughout the experiment to assess systemic toxicity. Mice were sacrificed on day 14 after ^125^I implantation for subsequent analyses, including tumor weight measurement, immunofluorescence staining, immunohistochemistry, and *in vivo* molecular imaging.

### 2.18 Immunofluorescence staining

Tumor tissues were harvested at day 14 after ^125^I implantation, fixed in 4% paraformaldehyde overnight, embedded in paraffin, and sectioned at a thickness of 4 μm. After deparaffinization and rehydration, antigen retrieval was performed in citrate buffer (pH 6.0) using heat-mediated retrieval. Sections were permeabilized with 0.3% Triton X-100 for 15 min and blocked with 5% bovine serum albumin (BSA) for 1 h at room temperature.

The sections were incubated overnight at 4°C with primary antibodies against calreticulin (CALR; Abcam, ab2907, 1:200) or HMGB1 (Abcam, ab18256, 1:200). After washing, samples were incubated with Alexa Fluor-conjugated secondary antibodies (Alexa Fluor 594 goat anti-rabbit IgG, Invitrogen, A11012, 1:500) for 1 h at room temperature in the dark. Nuclei were counterstained with DAPI (Beyotime, C1005). Fluorescence images were acquired using a fluorescence microscope, and representative fields were selected under identical exposure settings for all groups.

### 2.19 *In vivo* therapeutic study in the 4T1 bone metastasis model

The 4T1 bone metastasis model was established as previously described. Seven days post-tumor inoculation, mice were randomly assigned to five experimental groups (n=5 per group): model control; ¹²^5^I monotherapy (0.8 mCi/mouse); anti-PD-L1 antibody (5 mg/kg, every 3 days) combined with nab-paclitaxel (4 mg/kg, every 3 days); ^125^I treatment for 14 days followed by anti-PD-L1 combination; and anti-PD-L1 treatment for 14 days followed by ^125^I combination. Treatments were administered over a 4-week period. Tumor dimensions (length, a; width, b) were measured every 3 days, and tumor volume (TV) was calculated as: TV=(Length×Width^2^)/2.At the end of the study, mice were euthanized, and tumors were excised, photographed, and weighed to determine tumor growth inhibition.

### 2.20 Statistical analysis

All data are presented as mean ± standard error of the mean (SEM). Statistical analyses were performed using GraphPad Prism 8.0. For comparisons between two independent groups, unpaired two-tailed Student’s t-tests were applied. For comparisons among multiple groups, one-way or two-way analysis of variance (ANOVA) was used as appropriate, followed by *post hoc* multiple-comparison tests. Tukey’s multiple comparisons test was applied for one-way ANOVA analyses where all pairwise group comparisons were performed, whereas false discovery rate (FDR) correction was used when multiple hypotheses or time points were tested to control for type I error inflation. For experiments involving two independent variables, two-way ANOVA with FDR-adjusted multiple comparisons was conducted. Longitudinal data were analyzed using repeated-measures ANOVA with FDR correction applied to multiple comparisons. A two-sided P value<0.05 was considered statistically significant. Statistical significance is indicated as ns (*P*>0.05), * (*P* < 0.05), ** (*P* < 0.01), and *** (*P* < 0.001).

## 3 Result

### 3.1 ^125^I exhibits selective cytotoxicity against TNBC cells with preserved endothelial viability

**Figure 1:**
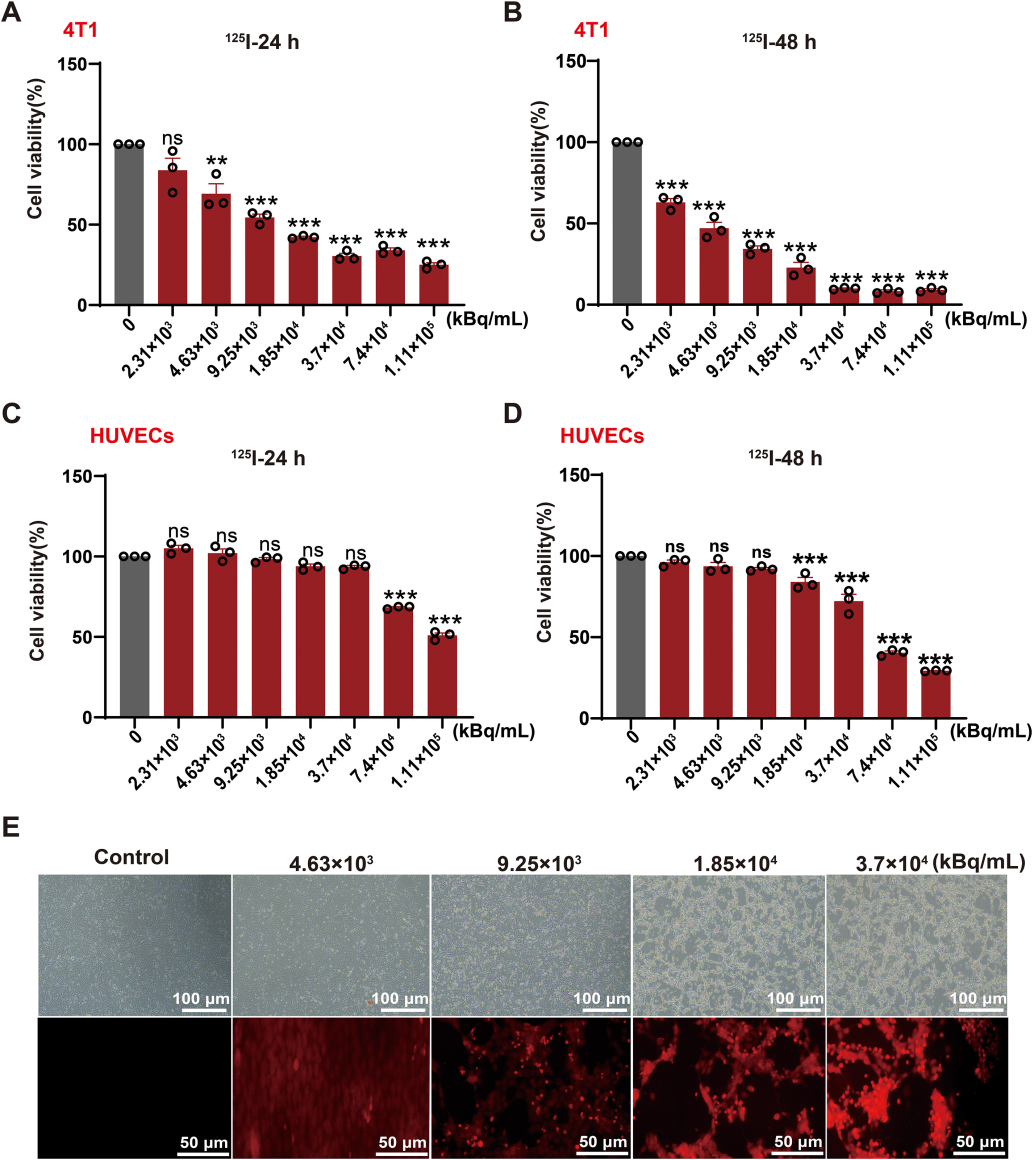
^125^I confers specific cytotoxicity against TNBC cells while sparing vascular endothelium. **(A, B)** Viability of 4T1 cells after 24h and 48h treatment with ^125^I. **(C, D)** Viability of HUVECs following 24h and 48h exposure to ^125^I. **(E)** Representative TUNEL staining of 4T1 cells following 12 h exposure to ¹²^5^I. The bright-field image (top, 10×) illustrates the overall cell morphology, whereas the fluorescence image (bottom, 20×) shows TUNEL-positive apoptotic cells (red). Scale bars: upper panels, 100 μm; lower panels, 50 μm. Quantitative data are presented as mean ± SEM from three independent experiments (n=3). Statistical significance was determined by one-way ANOVA with FDR-adjusted multiple comparisons. ns, *P*>0.05; *P < 0.05; **P < 0.01; ***P < 0.001.

We evaluated the direct cytotoxic effects of ¹²^5^I irradiation by measuring the viability of murine TNBC 4T1 cells and HUVECs across a range of doses and exposure durations using the CCK-8 assay. The viability of 4T1 cells was significantly inhibited by ¹²^5^I in a manner dependent on both dose and time (**Figures 1A, B**). In contrast, HUVECs demonstrated markedly greater resistance, maintaining high viability under the same treatment conditions (**Figures 1C, D**). TUNEL staining further confirmed that ¹²^5^I induced substantial apoptosis in 4T1 cells, with the effect scaling with dose (**Figure 1E**). Collectively, these *in vitro* findings establish a favorable therapeutic index for ¹²^5^I, characterized by a selectivity that demonstrates potent cytotoxicity against cancer cells while sparing the vascular endothelium, thereby ensuring treatment safety and positioning ¹²^5^I as a promising immune-priming modality based on the role of apoptosis in ICD initiation.

### 3.2 ^125^I triggers immunogenic cell death characterized by surface CALR translocation and HMGB1 secretion

**Figure 2:**
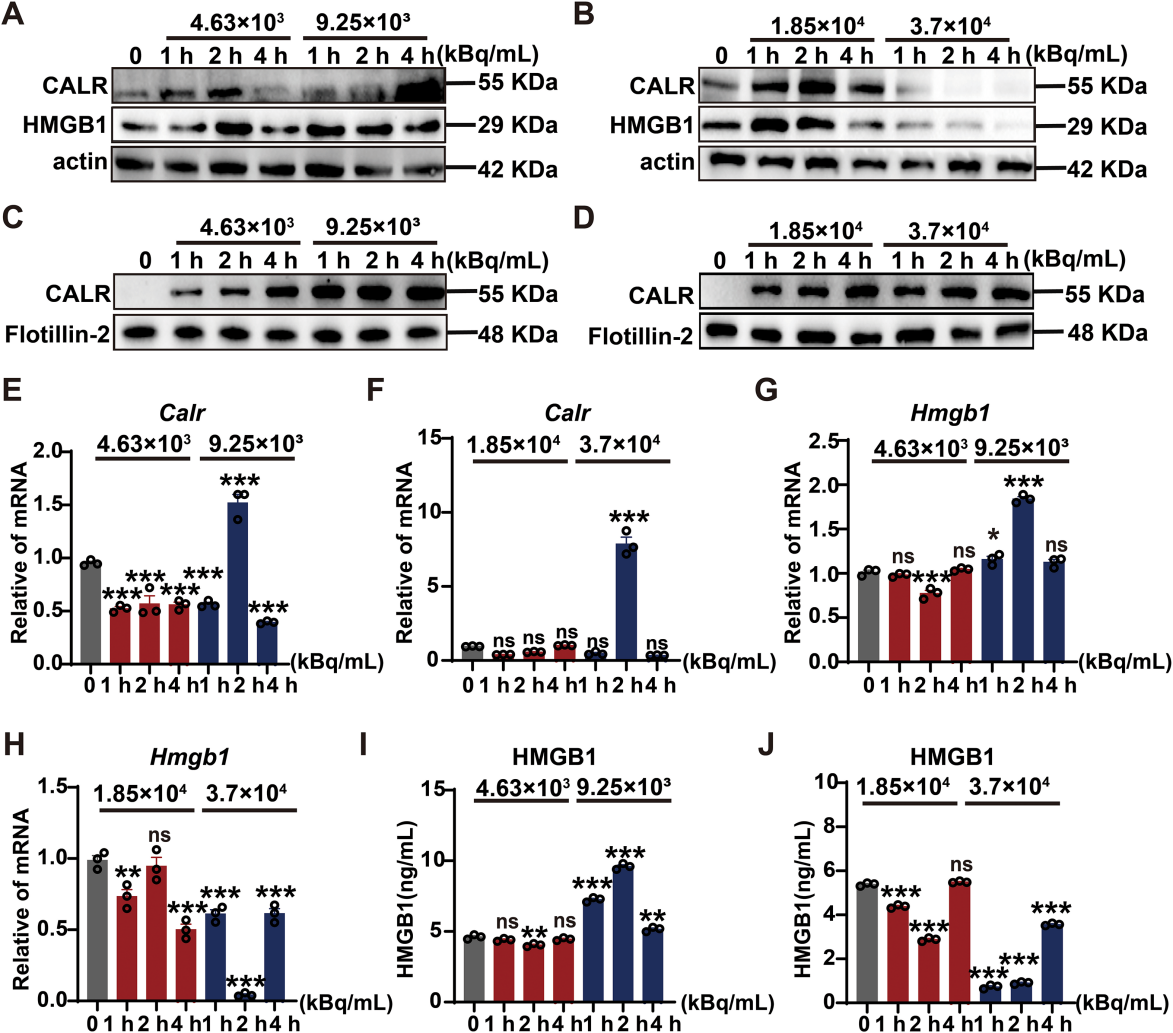
^125^I orchestrates immunogenic cell death through coordinated CALR exposure and HMGB1 release. **(A, B)** Western blot analysis of total CALR and HMGB1 protein expression in 4T1 cells following exposure to different ^125^I activity concentrations (4.63×10^3^, 9.25×10^3^, 1.85×10^4^, and 3.7×10^4^ kBq/mL) for the indicated durations (0, 1, 2, and 4 h). **(C, D)** Western blot analysis of membrane-associated CALR, with Flotillin-2 used as a plasma membrane marker, under the same treatment conditions. **(E, F)** qRT-PCR analysis of *Calr* mRNA expression **(G, H)** qRT-PCR analysis of *Hmgb1* mRNA expression. **(I, J)** ELISA quantification of extracellular HMGB1 levels in culture supernatants. Quantitative data are presented as mean ± SEM from three independent experiments (n=3). Statistical significance was determined using one-way or two-way ANOVA with FDR-adjusted multiple comparisons. ns, *P*>0.05; *P < 0.05; **P < 0.01; ***P < 0.001.

Given the selective cytotoxicity of ^125^I in 4T1 cells, we next examined whether this cell death displayed immunogenic features by analyzing two canonical DAMPs. Western blot analysis revealed that ^125^I irradiation significantly upregulated CALR and HMGB1 protein levels in a dose and time dependent manner, peaking at 2 h post-treatment (**Figures 2A, B**; **Supplementary Figure 1**),an early molecular event indicative of ICD. Critically, membrane fractionation assays confirmed substantial translocation of CALR from the endoplasmic reticulum to the cell surface (**Figures 2C, D**), establishing the essential “eat-me” signal for phagocytic recognition by antigen-presenting cells, a defining early step in ICD.

To further characterize the regulatory mechanisms underlying DAMP expression, we performed qRT-PCR, which showed significant transcriptional upregulation of both *Calr* and *Hmgb1* genes following ^125^I exposure (**Figures 2E–H**). This transcriptional reinforcement likely sustains the ICD phenotype beyond the initial protein response. Correspondingly, ELISA quantification demonstrated a progressive, dose dependent increase in extracellular HMGB1 release (**Figures 2I, J**), confirming active secretion of this danger signal that mediates dendritic cell activation and T−cell priming.

Together, these data indicate that ^125^I promotes ICD by enhancing CALR surface exposure and HMGB1 release while upregulating their transcription, thereby facilitating the initiation of antitumor immune responses. Notably, exposure to 9.25×10³kBq/mL ^125^I elicited the strongest ICD−related responses in 4T1 cells while preserving acceptable viability in HUVECs, providing a well−tolerated and potent dosing benchmark for subsequent *in vivo* studies aimed at activating antitumor immunity.

### 3.3 Local ^125^I brachytherapy achieves superior tumor control and safety over EBRT in bone-metastatic TNBC

**Figure 3:**
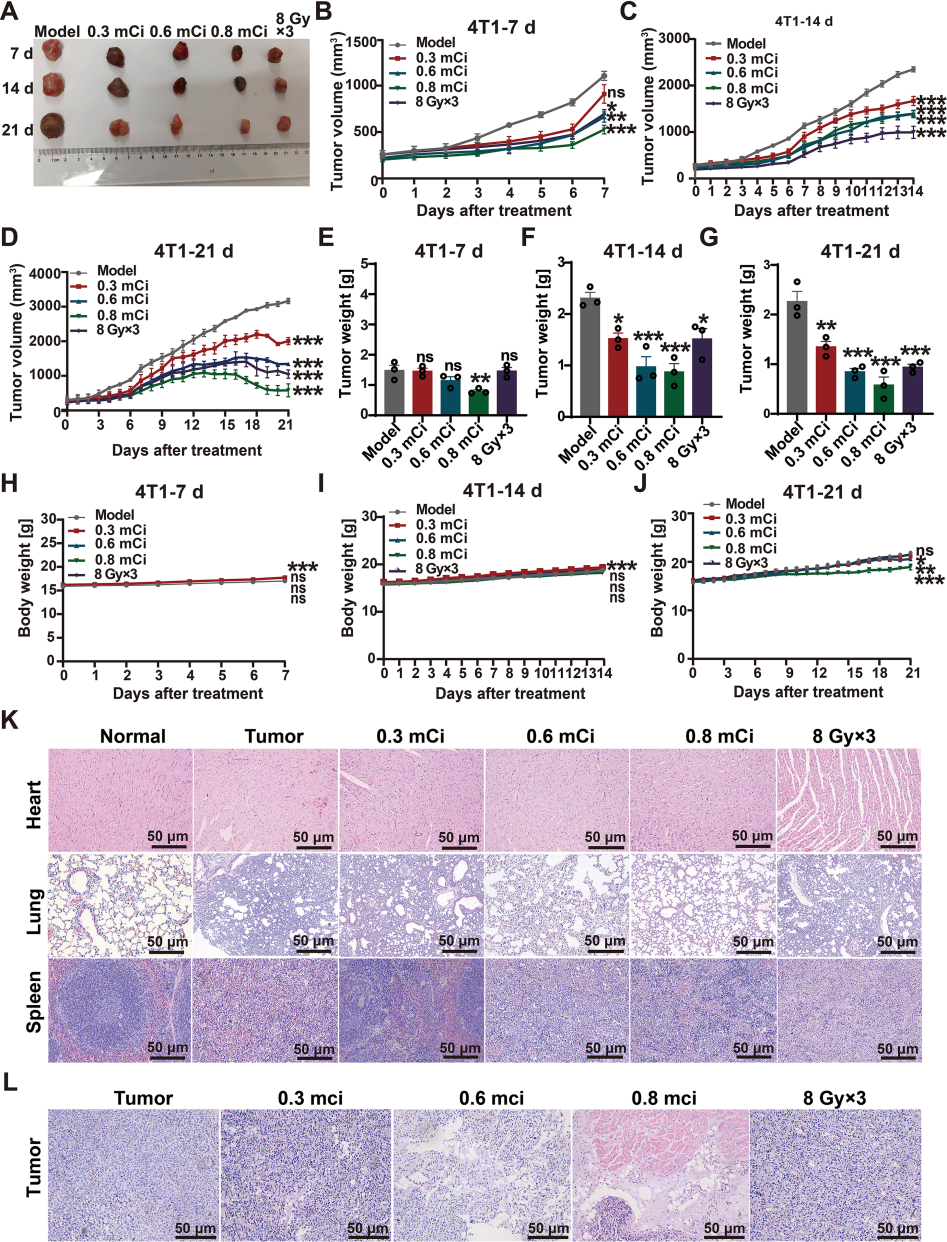
Local ^125^I brachytherapy achieves superior tumor control and safety over EBRT in bone-metastatic TNBC. **(A)** Representative images of excised bone metastatic tumors on days 7, 14, and 21 after treatment with ^125^I particles (0.3, 0.6, and 0.8 mCi) or external beam radiotherapy (EBRT; 8 Gy×3 fractions) (n=3 independent mice per group per time point). **(B–D)** Tumor volume progression over time assessed on days 7 (B; n=9 mice), 14 (C; n=6 mice), and 21 (D; n=3 mice). **(E–G)** Terminal tumor weights measured on days 7 **(E)**, 14 **(F)**, and 21 **(G)** (n=3 independent mice per group per time point). **(H–J)** Body weight monitoring during the treatment period on days 7 **(H)**, 14 **(I)**, and 21 **(J)** (n values as in B-D). **(K)** Representative H&E-stained histopathological images of major organs (heart, lung, and spleen) from each treatment group (n=3). **(L)** Representative H&E staining of tumor tissues from each treatment group (n=3).Quantitative data are presented as mean ± SEM. Statistical significance was determined using one-way or two-way ANOVA with false discovery rate (FDR)-adjusted multiple comparisons. ns, *P*>0.05; *P < 0.05; **P < 0.01; ***P < 0.001. The decreasing n values at later time points reflect the predefined experimental design, in which a subset of mice (n=3 per group per time point) was euthanized at each time point for histological and molecular analyses.

We evaluated the therapeutic efficacy of localized ^125^I brachytherapy for TNBC bone metastasis using a murine 4T1 model and compared outcomes with conventional EBRT. Local implantation of ^125^I seeds suppressed tumor growth in a manner dependent on both dose and time. The 0.8 mCi group exhibited the greatest inhibitory effect, outperforming EBRT (8 Gy×3) in reducing tumor volume (**Figures 3A–D**) and final tumor weight (**Figures 3E–G**). These findings establish the superior local control of continuous LDR irradiation over fractionated EBRT within this challenging metastatic niche. Critically, ^125^I brachytherapy showed an excellent safety profile. Throughout treatment, none of the ^125^I-treated groups displayed significant body weight loss (**Figures 3H–J**), indicating minimal systemic toxicity. Histopathological examination further confirmed favorable safety, revealing intact tissue morphology in major organs (heart, lung, spleen) of ^125^I-treated mice, whereas EBRT induced notable tissue damage (**Figure 3K**). This contrast highlights the tissue-sparing advantage of precisely localized brachytherapy over the broader irradiation field of EBRT.

At the tumor level, hematoxylin and eosin staining revealed extensive nuclear condensation and apoptotic morphology following high-dose (0.8 mCi) ^125^I treatment (**Figure 3L**), suggesting that tumor suppression involved direct induction of tumor cell death. Together, these results demonstrate that localized ^125^I brachytherapy exerts significant antitumor activity in the TNBC bone metastasis model while maintaining a safety profile superior to EBRT, providing a robust therapeutic foundation for subsequent evaluation of its immunomodulatory effects.

### 3.4 Sustained ^125^I brachytherapy induces ROS-associated immunogenic cell death and reshapes the immunosuppressive bone microenvironment

**Figure 4:**
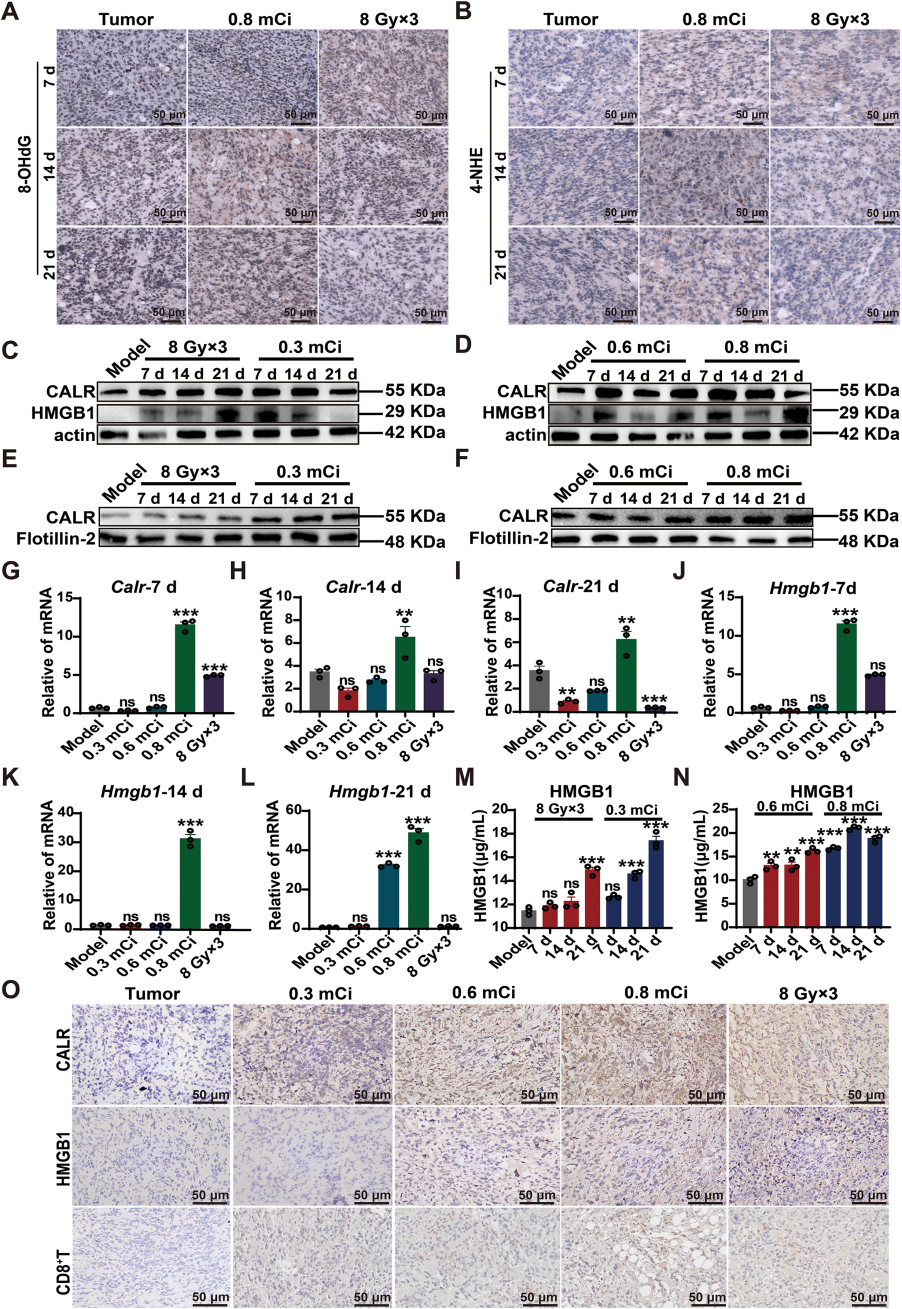
Sustained ^125^I brachytherapy induces ROS-associated immunogenic cell death and reshapes the immunosuppressive bone microenvironment. **(A, B)** Representative immunohistochemical (IHC) images showing the expression of the oxidative stress markers 8-OHdG (oxidative DNA damage) and 4-HNE (lipid peroxidation) in tumor tissues at days 7, 14, and 21 following different radiotherapy modalities. Scale bars, 50 µm. **(C, D)** Western blot analysis of total CALR and HMGB1 protein levels in tumor tissues after external beam radiotherapy (EBRT; 8 Gy×3 fractions) or local ^125^I implantation (0.3, 0.6, or 0.8 mCi) at days 7, 14, and 21. β-actin served as a loading control. **(E, F)** Western blot analysis of membrane-associated CALR under the same treatment conditions, with Flotillin-2 used as a membrane loading control. **(G–I)** Quantitative RT-PCR analysis of *Calr* mRNA expression in tumor tissues at the indicated time points following ^125^I treatment. **(J–L)** Quantitative RT-PCR analysis of *Hmgb1* mRNA expression under the same conditions. **(M, N)** ELISA quantification of extracellular HMGB1 levels in tumor tissues across treatment groups and time points. **(O)** Representative IHC images showing CALR, HMGB1, and tumor-infiltrating CD8^+^T cells in tumor tissues from different treatment groups. Scale bars, 50 µm. Quantitative data are presented as mean ± SEM from n=3 independent biological replicates per group. IHC images are representative of n=3 independent tumors per group. Statistical significance was determined using one-way or two-way ANOVA with FDR-adjusted multiple comparisons. ns, not significant; *P < 0.05; **P < 0.01; ***P < 0.001.

To investigate the immunomodulatory effects of ^125^I brachytherapy in bone-metastatic TNBC, we compared intratumoral oxidative stress and downstream immunogenic responses induced by ^125^I and EBRT. Immunohistochemical analyses revealed that 0.8 mCi ^125^I implantation induced markedly higher and more sustained oxidative DNA and lipid damage, as indicated by progressive accumulation of 8-OHdG and 4-HNE, peaking at day 14 post-treatment. In contrast, EBRT (8 Gy×3) elicited only a transient oxidative response that declined after day 7 (**Figures 4A, B**; **Supplementary Figures 2A, B**).

Consistent with sustained oxidative stress, ^125^I brachytherapy induced robust activation of ICD signaling. CALR and HMGB1 protein expression increased in a dose-dependent manner following ^125^I treatment and was significantly higher than that induced by EBRT (**Figures 4C, D**; **Supplementary Figures 2C-F**). Subcellular fractionation further demonstrated enhanced redistribution of CALR to the plasma membrane in tumors exposed to 0.8 mCi ^125^I, indicative of increased surface exposure of this canonical “eat-me” signal (**Figures 4E, F**; **Supplementary Figures 2G, H**).

At the transcriptional and secretory levels, *Calr* and *Hmgb1* mRNA expression remained persistently elevated up to 21 days after ^125^I treatment, accompanied by sustained extracellular HMGB1 release, whereas EBRT induced comparatively modest and short-lived changes (**Figures 4G–N**). Importantly, tumors treated with ^125^I exhibited increased intratumoral CD8^+^T-cell infiltration that spatially co-localized with regions of elevated CALR and HMGB1 expression, whereas EBRT-treated tumors showed sparse immune infiltration (**Figure 4O**; **Supplementary Figures 2I–K**). Collectively, these data demonstrate that continuous low-dose-rate ^125^I brachytherapy establishes a sustained oxidative and immunogenic tumor milieu, characterized by persistent ICD signaling and enhanced CD8^+^T-cell infiltration, thereby reshaping the immunosuppressive bone metastatic microenvironment more effectively than EBRT.

### 3.5 Temporal transcriptomic profiling reveals a progressive stress-response program associated with sustained ICD Induction

**Figure 5:**
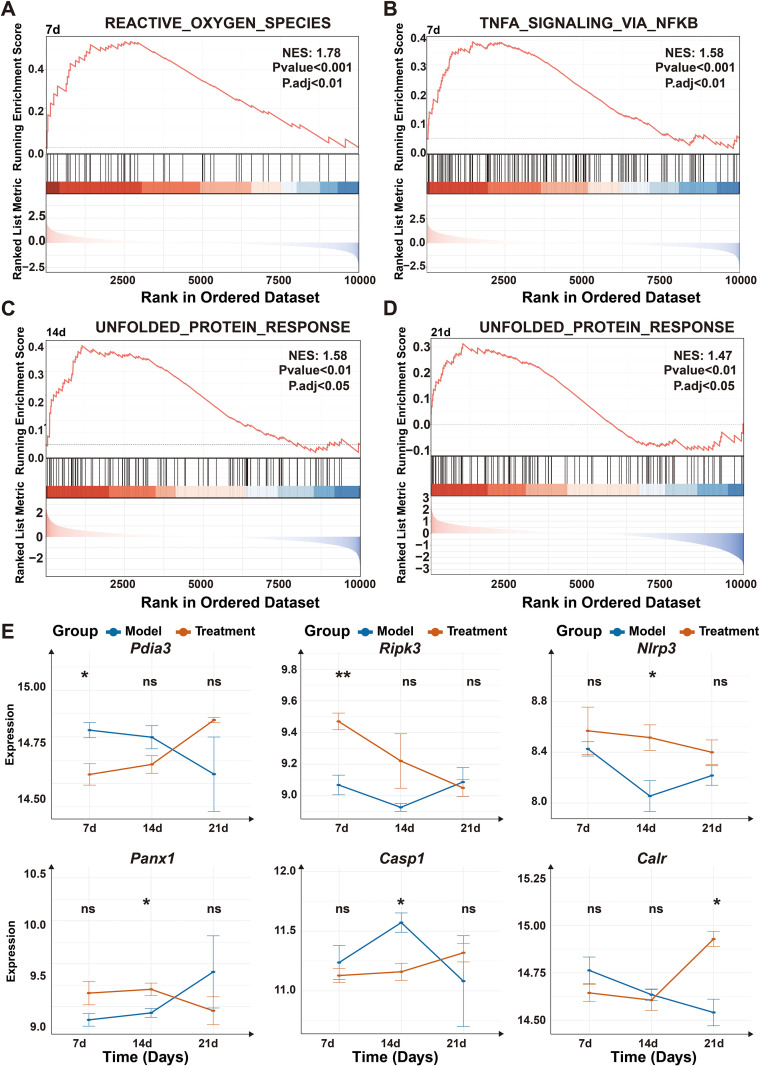
Temporal transcriptomics unravel a progressive ICD program driven by unfolded protein response. **(A, B)** Gene Set Enrichment Analysis (GSEA) showing significant enrichment of the HALLMARK_REACTIVE_OXYGEN_SPECIES **(A)** and HALLMARK_TNFA_SIGNALING_VIA_NFKB **(B)** pathways in ^125^I-treated tumors compared with model controls at day 7 post-treatment **(C, D)** GSEA demonstrating enrichment of the HALLMARK_UNFOLDED_PROTEIN_RESPONSE pathway at day 14 **(C)** and day 21 **(D)** following ^125^I treatment. Normalized Enrichment Score (NES) and false discovery rate–adjusted q values (FDR) are indicated for each pathway. **(E)** Temporal expression profiles of representative ICD-and DAMP-related genes (*Pdia3*, *Ripk3*, *Nlrp3*, *Panx1*, *Casp1*, and *Calr*) in model control and ^125^I-treated tumors at days 7, 14, and 21, derived from bulk RNA sequencing data. Expression values represent DESeq2-normalized log2 counts. Statistical comparisons between treatment and model groups at each time point were performed using the DESeq2 Wald test with FDR correction (Benjamini-Hochberg).Data are presented as mean ± SEM (n=3 biological replicates per group per time point). ns, not significant; *P < 0.05; **P < 0.01; ***P < 0.001.

To elucidate the molecular programs underlying the sustained immunogenic effects of ^125^I brachytherapy, we performed RNA sequencing of tumor tissues collected at days 7, 14, and 21 following treatment. Gene set enrichment analysis (GSEA) revealed a temporally coordinated activation of stress- and immunity-related pathways in ^125^I-treated tumors (**Figures 5A–D**).

At the early stage (day 7), pathways associated with oxidative stress and inflammatory signaling were prominently enriched, including the REACTOME_REACTIVE_OXYGEN_SPECIES_PATHWAY (NES = 1.78, adjusted *P* < 0.01) and HALLMARK_TNFA_SIGNALING_VIA_NFKB (NES = 1.58, adjusted *P* < 0.01). This transcriptional profile is consistent with an acute cellular stress response characterized by elevated ROS signaling and pro-inflammatory priming of the tumor microenvironment.

At later time points, transcriptomic signatures indicative of unresolved cellular stress became dominant. Notably, the HALLMARK_UNFOLDED_PROTEIN_RESPONSE (UPR) pathway remained significantly enriched at both day 14 (NES = 1.58, adjusted *P* < 0.05) and day 21 (NES = 1.47, adjusted *P* < 0.05), indicating persistent endoplasmic reticulum stress during prolonged low-dose-rate irradiation. Consistent with sustained UPR activation, DAMP-related genes exhibited a structured temporal expression program(**Figure 5E**). Early induction of *Ripk3* (*P* < 0.01) accompanied by suppression of *Pdia3* (*P* < 0.05) suggested initiation of immunogenic stress responses, followed by increased expression of *Panx1* and *Nlrp3* at day 14 (*P* < 0.05), indicative of enhanced DAMP release capacity. By day 21, significant upregulation of *Calr* (*P* < 0.05) was observed, corresponding to a late-stage immunogenic phenotype characterized by efficient calreticulin exposure. This cascade demonstrates that ^125^I brachytherapy induces a temporally coordinated and durable ICD program, effectively bridging early stress responses with late-phase immune activation through its continuous LDR delivery profile. These transcriptomic findings provide molecular evidence for the sustained immunogenic capacity of ^125^I seeds, highlighting their fundamental advantage over the transient immune responses induced by EBRT.

### 3.6 ROS is functionally required for ^125^I-induced ICD and antitumor immune activation

To determine whether ROS signaling is functionally required for ^125^I-induced ICD, pharmacological ROS inhibition was performed in the 4T1 bone metastasis model. Based on the temporal kinetics of ROS accumulation and ICD marker expression (**Figure 4**), day 14 was selected for downstream functional analyses.

**Figure 6:**
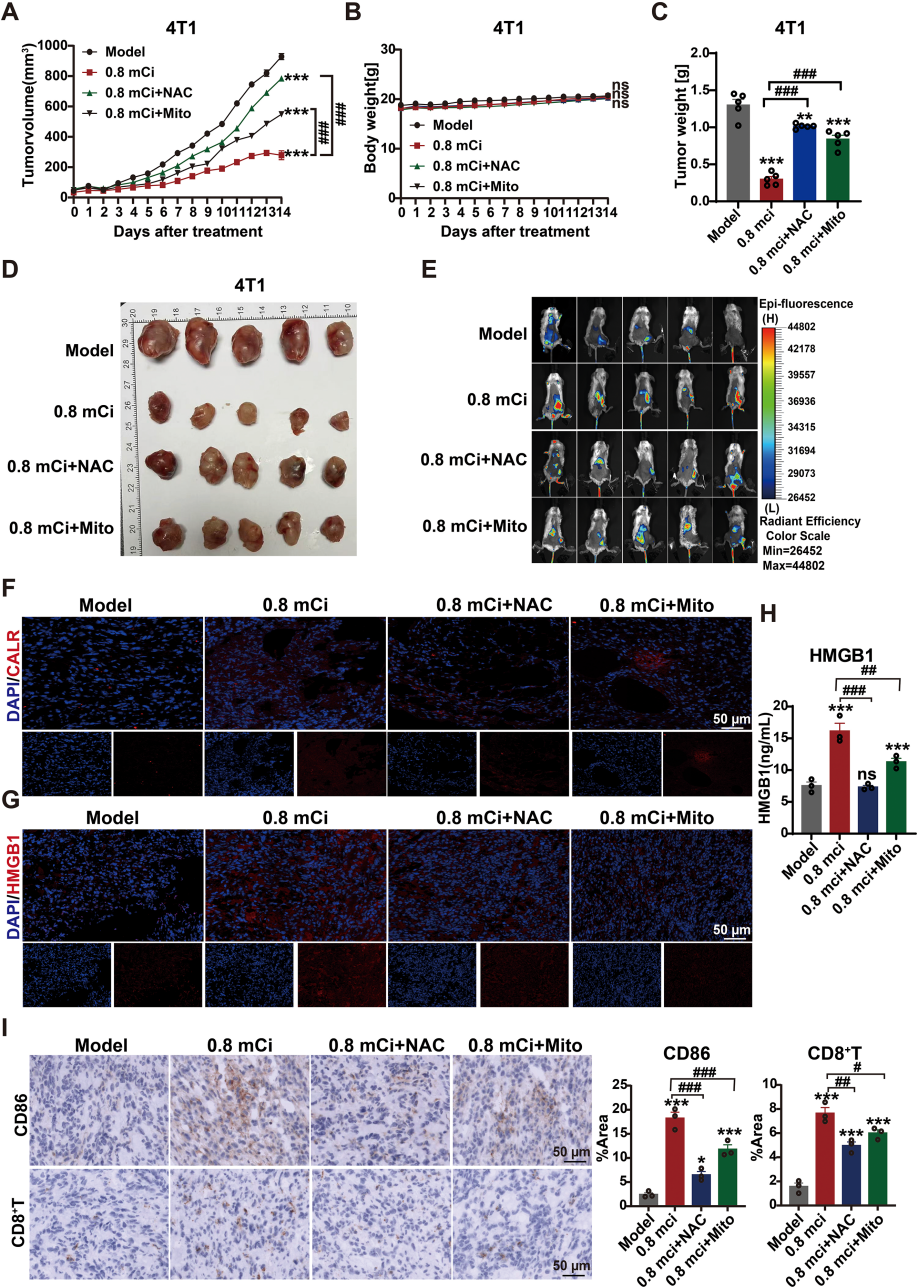
ROS inhibition attenuates ^125^I brachytherapy-induced ICD and antitumor immune activation. **(A)** Tumor growth curves of 4T1 tumor-bearing mice treated with 0.8 mCi ^125^I brachytherapy alone or in combination with the ROS scavenger N-acetylcysteine (NAC) or the mitochondria-targeted antioxidant MitoQ. **(B)** Body weight monitoring of mice under the indicated treatment conditions. **(C)** Tumor weights measured at day 14 after treatment. **(D)** Representative images of excised tumors collected at day 14 from each treatment group. **(E)** Representative *in vivo* CALR-targeted fluorescence imaging of tumors at day 14 following ^125^I brachytherapy with or without ROS inhibition. **(F)** Representative immunofluorescence images showing CALR surface exposure in tumor tissues (CALR, red; DAPI, blue). **(G)** Representative immunofluorescence images showing HMGB1 expression and release in tumor tissues (HMGB1, red; DAPI, blue). **(H)** ELISA-based quantification of extracellular HMGB1 release in tumor tissues at day 14 after treatment. **(I)** Representative immunohistochemical staining and quantitative analysis of CD86 and CD8^+^T cells in tumor tissues at day 14 after treatment. Data are presented as mean ± SEM (n=5 mice per group). Tumor growth curves in **(A)** were analyzed using two-way ANOVA. All other quantitative analyses were performed using one-way ANOVA followed by Tukey’s multiple comparisons test.*P < 0.05, **P < 0.01, ***P < 0.001 versus the Model group;^#^*P* < 0.05, ^##^*P* < 0.01, ^###^*P* < 0.001 versus the 0.8 mCi ^125^I treatment group; ns, not significant.

Implantation of 0.8 mCi ^125^I seeds significantly suppressed tumor growth, whereas co-administration of the pan-ROS scavenger NAC largely abrogated this antitumor effect. In contrast, the mitochondria-targeted antioxidant MitoQ partially attenuated tumor suppression (**Figure 6A**). Tumor weight measurements at day 14 mirrored these findings, while body weight remained comparable among groups, indicating minimal systemic toxicity (**Figures 6B–D**).

At the level of ICD execution, *in vivo* imaging using the CALR-targeted probe KLGFFKRC-Mal-Cy5.5 revealed robust tumor-associated fluorescence following ^125^I brachytherapy, which was markedly reduced by NAC and moderately diminished by MitoQ (**Figure 6E**). Consistently, immunofluorescence analysis demonstrated that ROS scavenging impaired CALR surface exposure and reduced HMGB1 release induced by ^125^I irradiation (**Figures 6F–G**). ELISA further confirmed that ^125^I-induced elevation of circulating HMGB1 was nearly abolished by NAC and partially attenuated by MitoQ (**Figure 6H**).

Functionally, inhibition of ROS-dependent ICD was accompanied by impaired antitumor immune activation. ^125^I brachytherapy markedly increased intratumoral CD8^+^T-cell infiltration and CD86 expression, both of which were strongly reduced by NAC and partially diminished by MitoQ (**Figure 6I**).Together, these results establish sustained ROS signaling as a functional prerequisite for ^125^I-induced ICD and subsequent antitumor immune activation. The differential effects of NAC and MitoQ indicate that mitochondrial ROS contributes to, but does not exclusively account for, the immunogenic consequences of continuous low-dose-rate ^125^I irradiation.

### 3.7 CALR-targeted molecular imaging enables spatiotemporal visualization of ROS-dependent ICD induced by ^125^I brachytherapy

Based on the ROS-dependent CALR exposure established in Section 3.6, we next evaluated whether CALR-targeted molecular imaging could serve as a noninvasive approach to visualize the spatiotemporal dynamics of immunogenic cell death induced by ^125^I brachytherapy. For *in vitro* validation, FITC-labeled KLGFFKR peptide was used to detect CALR exposure on tumor cells following ^125^I treatment. For *in vivo* imaging, a near-infrared probe (KLGFFKRC-Mal-Cy5.5) was employed to enable real-time visualization of CALR-expressing tumor regions in bone-metastatic lesions.

**Figure 7:**
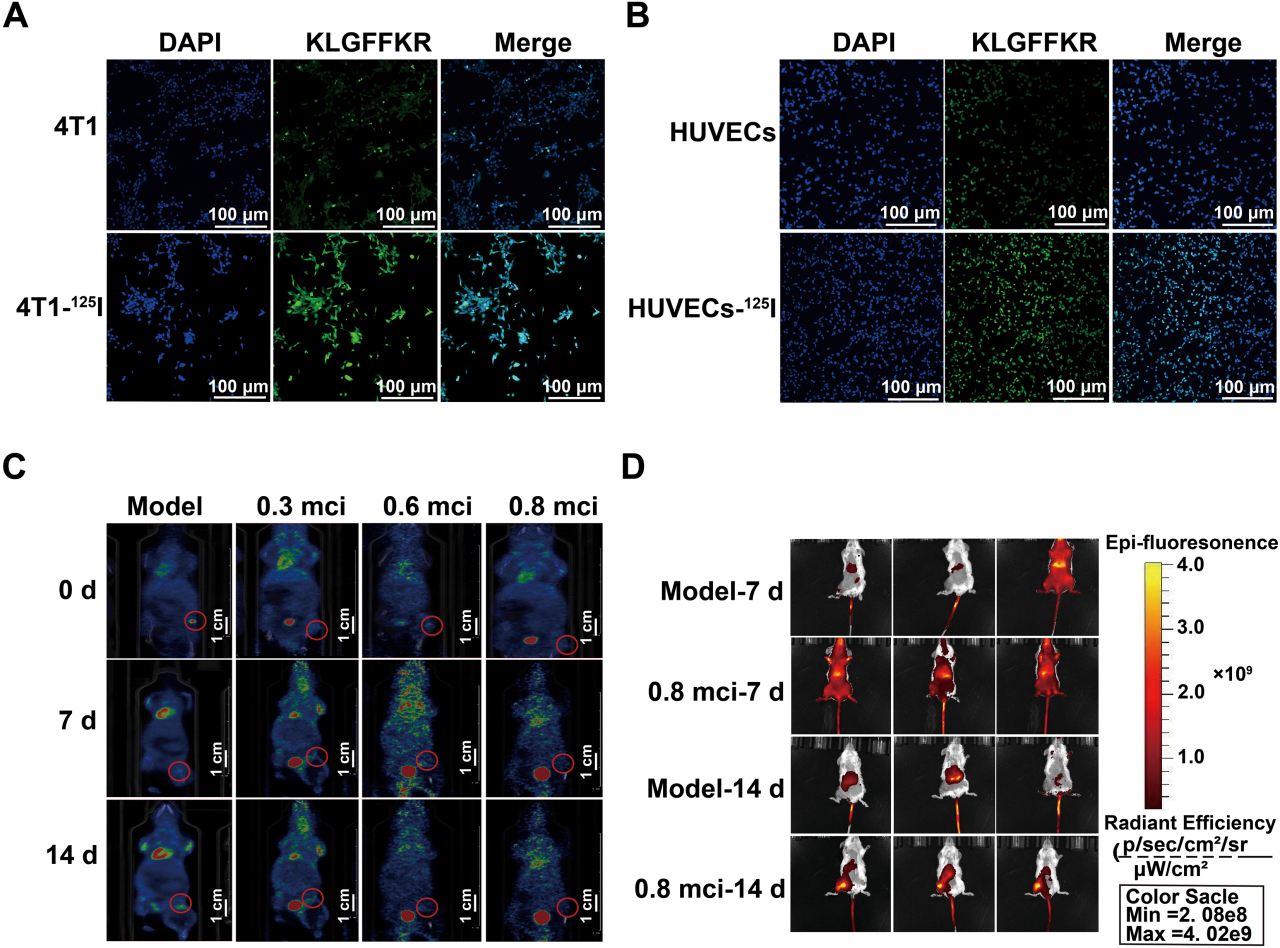
A novel CALR-targeted molecular probe enables real-time visualization of brachytherapy-induced ICD. **(A, B)** Immunofluorescence staining showing the cellular uptake of the fluorescently labeled KLGFFKR peptide in 4T1 cells **(A)** and HUVECs **(B)** after ^125^I treatment. Nuclei were stained with DAPI (Green), and the peptide signal (pseudo-colored green) is shown. The merged images show the overlay of the peptide signal with nuclei. Scale bar: 100 μm. **(C)** Representative PET/CT maximum intensity projection (MIP) images showing tumor metabolic activity following intravenous injection of [¹^8^F]FDG. Arrows indicate the tumor location in the tibia. **(D)** *In vivo* immunofluorescence imaging showing the cellular accumulation of the KLGFFKRC-Mal-Cy5.5 probe in the tumor site after intravenous injection. Images are representative of n=3 independent experiments.

*In vitro*, ^125^I-irradiated 4T1 cells exhibited characteristic features of immunogenic stress, including nuclear condensation and prominent CALR translocation to the plasma membrane, whereas control cells displayed predominantly cytoplasmic CALR localization (**Figures 7A, B**). Notably, the CALR-targeted probe showed minimal binding to non-malignant HUVECs, supporting tumor-specific detection of ICD-associated stress.

Based on integrated molecular and functional analyses (Sections 3.4-3.6), day 14 was selected for *in vivo* imaging. Small-animal PET/CT demonstrated reduced ^18^F-FDG uptake following ^125^I implantation, consistent with suppressed tumor metabolism (**Figure 7C**). Importantly, near-infrared imaging revealed robust and tumor-specific accumulation of KLGFFKRC-Mal-Cy5.5 in ^125^I-treated lesions, with fluorescence intensity closely correlating with intratumoral CALR expression (**Figure 7D**).

Collectively, these imaging data provide direct spatiotemporal evidence that^125^I brachytherapy induces sustained, ROS-dependent CALR exposure *in vivo*. By integrating functional perturbation with molecular imaging using the same CALR-targeted probe platform, this approach establishes a noninvasive framework for real-time visualization of ICD dynamics and for guiding rational integration of brachytherapy with immunotherapeutic strategies.

### 3.8 ^125^I brachytherapy elicits systemic immunity to drive abscopal regression

**Figure 8:**
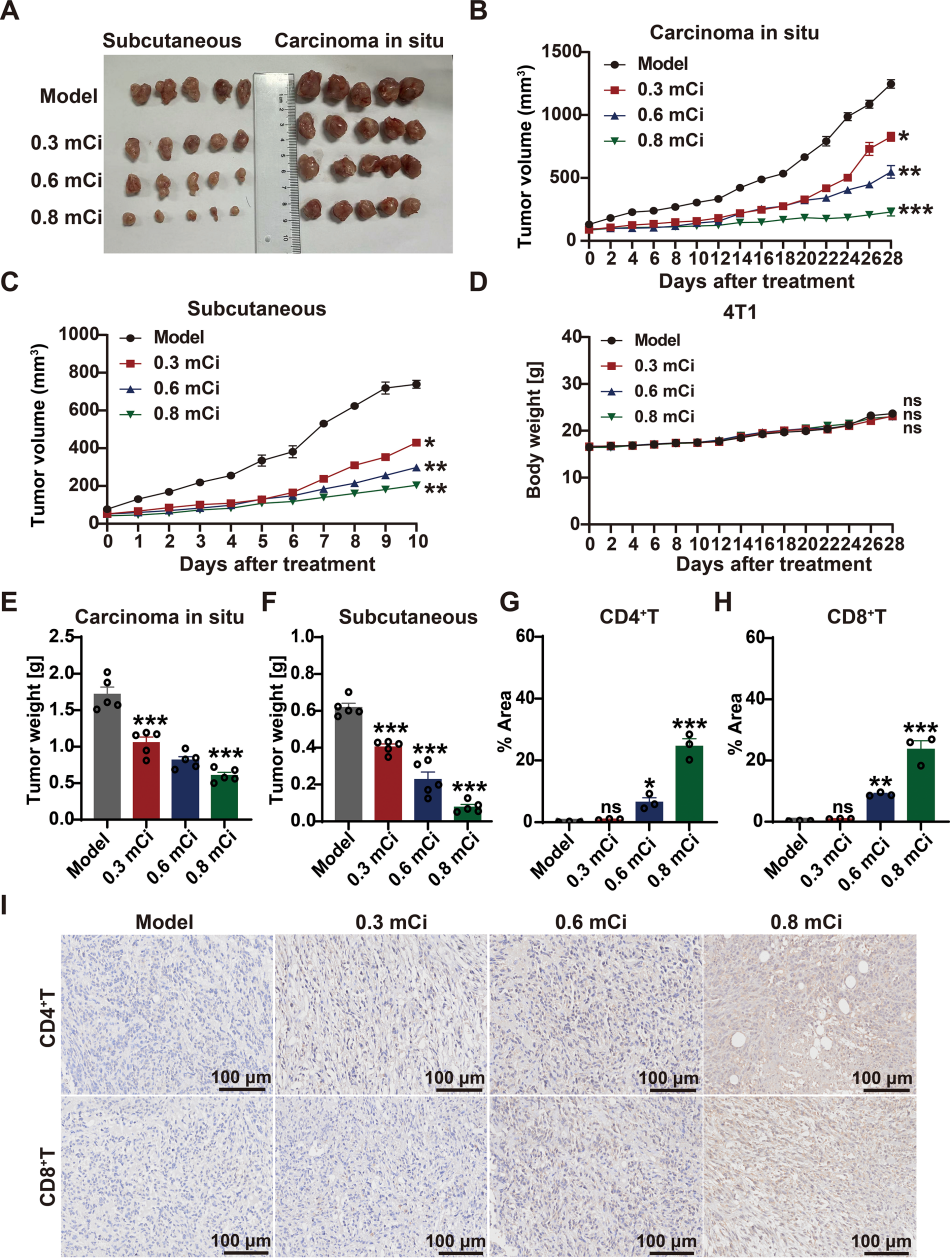
^125^I brachytherapy elicits systemic immunity to drive abscopal regression. **(A)** Representative photographs of primary bone metastatic tumors (right) and distant subcutaneous tumors (left) from each treatment group at the study endpoint. **(B)** Growth curves of primary bone metastatic tumors following treatment with different doses of ¹²^5^I brachytherapy. **(C)** Growth curves of distant subcutaneous tumors during the treatment period. **(D)** Body weight changes of mice during the treatment period. **(E)** Final weights of primary bone metastatic tumors at the study endpoint. **(F)** Final weights of distant subcutaneous tumors at the study endpoint. **(G, H)** Quantitative analysis of CD4^+^ **(G)** and CD8^+^ **(H)** T cell infiltration in distant subcutaneous tumors from each treatment group. **(I)** Representative IHC images of CD4^+^and CD8^+^T cells in distant subcutaneous tumors corresponding to the quantification shown in **(G)** and **(H)**. Scale bar, 100 μm. For IHC quantification, the value for each mouse represents the average obtained from three randomly selected, non-overlapping high-power fields. Data are presented as mean ± SEM (n=5 biologically independent mice per group). Statistical significance was determined using one-way ANOVA followed by Tukey’s multiple comparisons test. ns, not significant; *P < 0.05; **P < 0.01; ***P < 0.001. The images shown in **(I)** are representative of the immune infiltration observed across all samples.

To determine whether local ^125^I brachytherapy could elicit systemic antitumor immunity, we established a bilateral tumor model with primary bone metastases and secondary distal subcutaneous tumors. Local implantation of ^125^I seeds effectively suppressed the growth of both primary and distal tumors in a dose dependent manner (**Figures 8A–C**). The 0.8 mCi group demonstrated the most pronounced inhibitory effects, with tumor volumes and weights significantly lower than those in the 0.3 mCi, 0.6 mCi, and control groups (**Figures 8E, F**), confirming a robust abscopal effect specifically associated with the optimal therapeutic dose. Throughout the treatment period, no significant body weight loss was observed (**Figure 8D**), indicating excellent systemic tolerability and supporting the clinical translational potential of this approach.

IHC analysis of the distal subcutaneous tumors provided mechanistic insights into this abscopal effect. We observed an increase in the infiltration of both CD4^+^T and CD8^+^T cells in the 0.8 mCi group (**Figures 8G–I**), demonstrating that the systemic tumor suppression is mediated by the activation and recruitment of immune effector cells. This spatial pattern of T cell infiltration, specifically in non irradiated distal lesions, provides direct evidence that the abscopal effect is initiated by therapy induced ICD in the primary tumor, leading to systemic T cell priming and trafficking. Collectively, these findings establish that local ^125^I brachytherapy not only controls primary tumor growth but also drives a potent systemic antitumor immune response, effectively overcoming the immunosuppressive constraints of the bone metastatic microenvironment and validating its capacity to induce meaningful abscopal responses.

### 3.9 A priming sequence of ^125^I brachytherapy is a prerequisite for synergistic anti-PD-L1 checkpoint blockade

To establish the optimal therapeutic sequence, we compared two regimens for combining ^125^I brachytherapy with anti-PD-L1 immunotherapy and nab-paclitaxel in the 4T1 TNBC bone metastasis model. Mice received either 0.8 mCi ^125^I brachytherapy for 14 days followed by anti-PD-L1 antibody plus nab-paclitaxel for another 14 days (^125^I-first regimen),or the reverse sequence (immunochemotherapy-first regimen).

**Figure 9:**
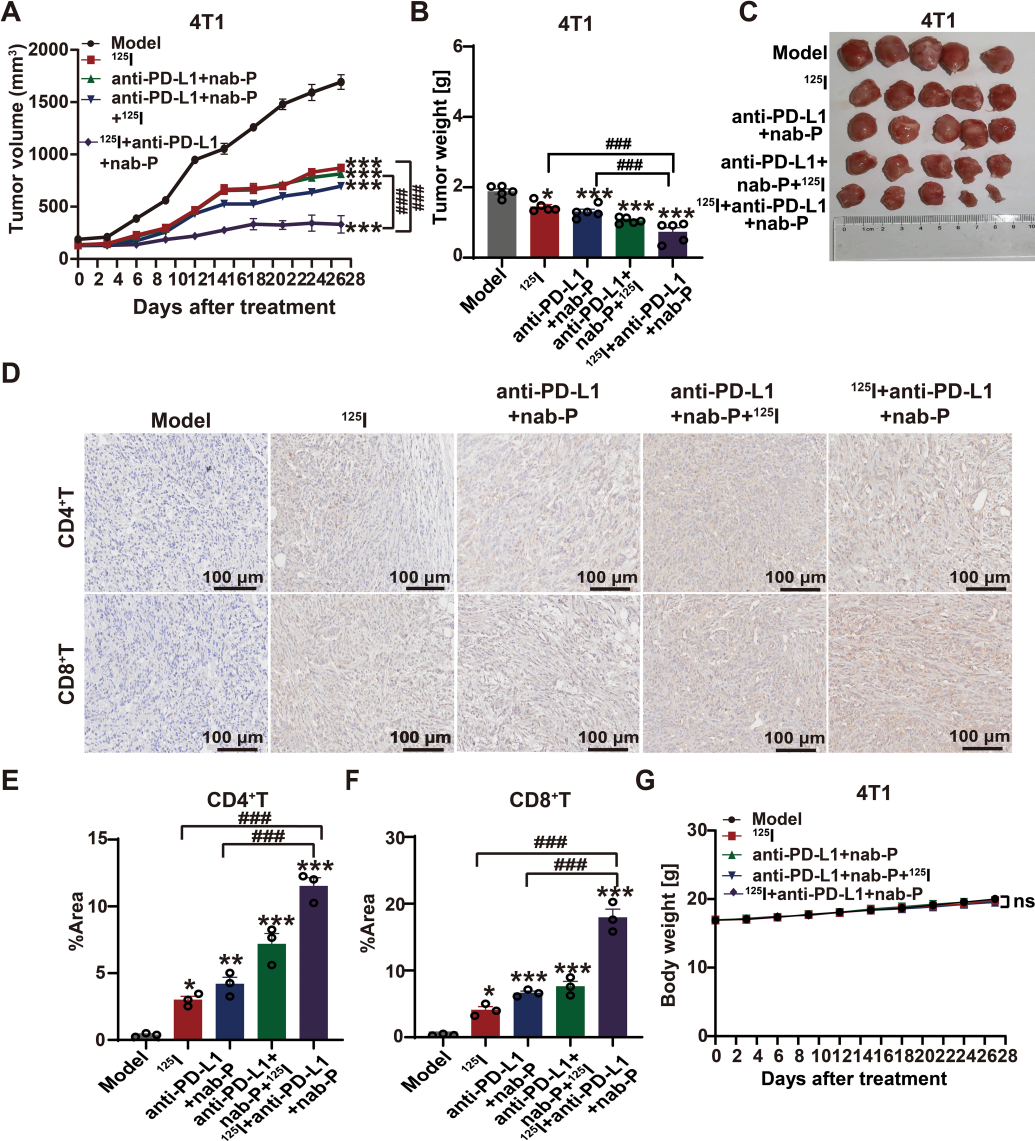
A priming sequence of ^125^I brachytherapy is a prerequisite for synergistic anti-PD-L1 checkpoint blockade. **(A)** Tumor growth curves of 4T1 tumor-bearing mice subjected to different treatment regimens, including monotherapies and sequential combination strategies. **(B)** Quantification of final tumor weights at the study endpoint. **(C)** Representative photographs of excised tumors from each treatment group at the study endpoint. **(D)** Representative IHC staining of CD4^+^and CD8^+^T cells in tumor tissues across the indicated treatment groups. Scale bar, 100 μm. **(E)** Quantitative analysis of CD4^+^T cell infiltration. **(F)** Quantitative analysis of CD8^+^T cell infiltration. **(G)** Body weight changes of mice during the treatment period. For IHC quantification in **(E)** and **(F)**, values for each mouse represent the average obtained from three randomly selected, non-overlapping high-power fields. Data are presented as mean ± SEM (n=5 biologically independent mice per group). Tumor growth curves were analyzed using two-way ANOVA, while endpoint measurements were analyzed using one-way ANOVA followed by Tukey’s multiple comparisons test. *P < 0.05, **P < 0.01, ***P < 0.001 versus the control group; ^#^*P* < 0.05, ^##^P<0.01, ^###^*P* < 0.001 indicate comparisons between combination therapy and the corresponding monotherapy groups; ns, not significant. Images shown in **(D)** are representative of the observations across all samples.

The triple combination therapy inhibited tumor growth more effectively than any monotherapy, evidenced by reduced tumor volumes (**Figures 9A–C**) and maintained systemic tolerability (**Figure 9G**).Critically, initiating treatment with ^125^I brachytherapy produced markedly stronger tumor suppression than the reverse sequence, establishing the temporal dependence of this synergistic effect. Mechanistically, IHC analysis revealed that the ^125^Ifirst regimen induced the most robust infiltration of both CD4^+^and CD8^+^T cells into tumor tissues (**Figure 9D–F**),directly demonstrating the priming effect of brachytherapy on the tumor immune microenvironment. The 14day priming interval was selected based on time course data showing that, by this point, ^125^I treatment had already induced significant immunogenic cell death (evidenced by elevated CALR and HMGB1),activated key pathways including the unfolded protein response, and initiated substantial CD8^+^T cell infiltration. This creates an immune−favorable context for checkpoint blockade. While transcriptomic profiles indicated further immune maturation beyond day 14,initiating immunotherapy at this juncture balances sufficient immune priming with the clinical imperative for timely therapeutic intervention.

## 4 Discussion

**Figure 10:**
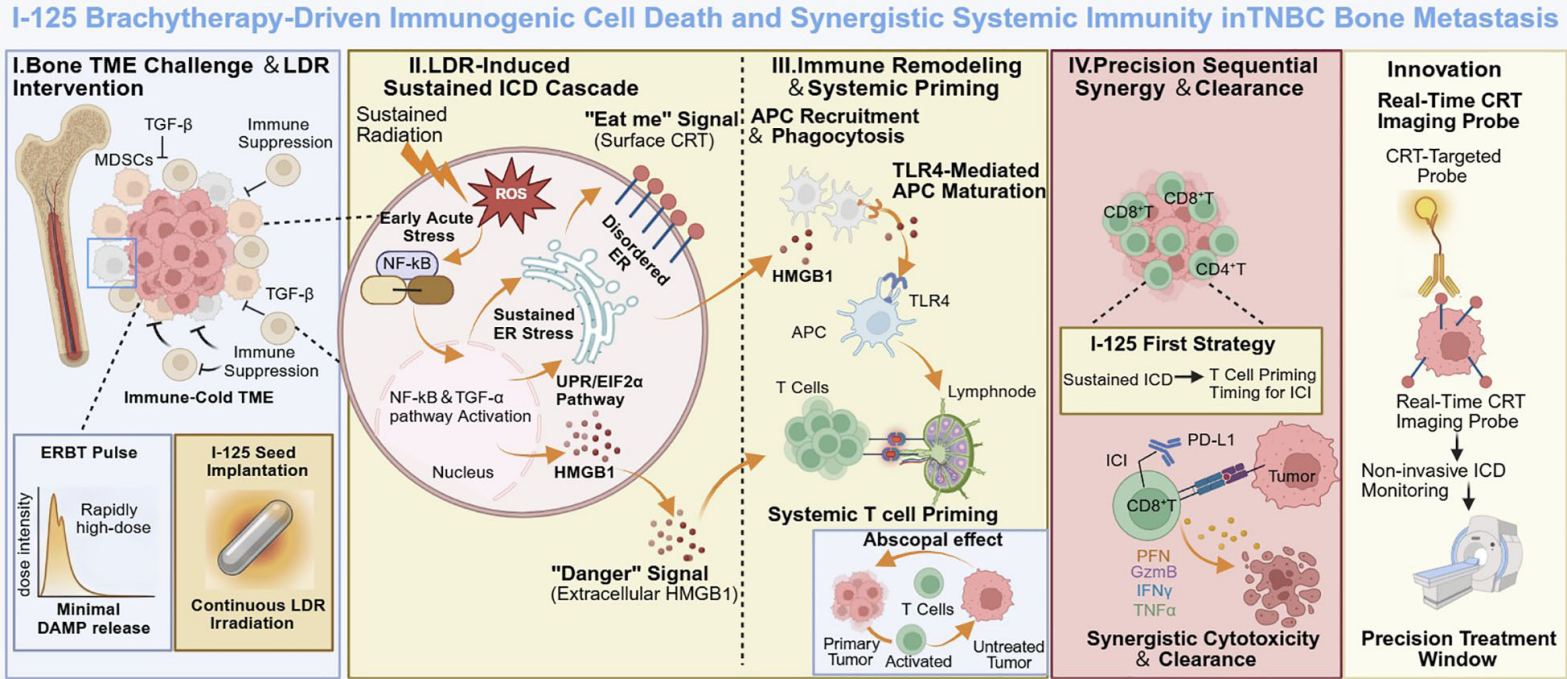
Mechanistic overview of ^125^I brachytherapy–driven sustained immunogenic cell death and synergistic systemic immunity in bone-metastatic TNBC. Continuous low dose rate irradiation from ^125^I seeds generates persistent ROS and prolonged ER stress, leading to stepwise exposure of calreticulin and release of HMGB1.These immunogenic cell death signals promote APC recruitment, TLR4-dependent maturation, and systemic T cell priming. The resulting immune hot microenvironment enhances the response to anti PD-L1 therapy and drives synergistic CD8 positive T cell mediated tumor clearance, including abscopal effects.

The treatment of bone metastatic TNBC is confronted by three core challenges: profound immunosuppression limiting CD8^+^T cell infiltration, the inability of conventional EBRT to induce sustained immune activation, and consequent primary resistance to ICIs. This study demonstrates that continuous LDR brachytherapy with ¹²^5^I seeds directly addresses these barriers. By integrating *in vitro* experiments, an orthotopic bone metastasis model, transcriptomic analysis, and ICD targeted molecular imaging, we show that ¹²^5^I induces a sustained and progressive ICD program over 7 to 21 days. This converts immunologically cold bone tumors into hot states, achieves superior local control and immune activation compared to EBRT, and primes the tumor microenvironment for subsequent ICIs (**Figure 10**).

Our study directly addresses the long-standing question of whether optimizing the temporal dynamics of radiotherapy can overcome the persistent immunosuppressive microenvironment in bone metastases. The bone metastatic niche is primarily governed by TGF−β–mediated immunosuppression (26), and current strategies aimed at reversing this, such as MDSC depletion, TGF−β blockade, or cancer vaccines, face considerable limitations in efficacy, durability, and safety, largely due to insufficiently strong and sustained immune activation (10, 27–29) Conventional EBRT induces only transient immune stimulation, which is poorly matched to the chronically immunosuppressive milieu of bone metastases. Preclinical studies have shown that although T cell infiltration peaks early following EBRT, it rapidly declines thereafter, and innate immune signals are similarly short-lived (30–32). Recent work further demonstrates that the immunogenic consequences of radiotherapy are significantly influenced by dose, dose rate, and fractionation schedule, which collectively regulate antigen presentation, immune cell recruitment, and systemic antitumor responses. Appropriately designed single-dose or fractionated regimens can effectively activate dendritic cells and prime effector T cells, enhancing both the quality and durability of the immune response (33, 34).In this context, low-fractionated radiotherapy combined with immune modulators has been shown to markedly improve immune infiltration and clinical outcomes in metastatic solid tumors (35, 36). However, such strategies remain highly dependent on the short-lived immune activation window induced by EBRT, which is insufficient to fully overcome the TGF−β–driven chronic immunosuppression in bone metastases. Recently, approaches combining TGF−β inhibitors or bispecific TGF−β/PD−L1 antibodies have shown partial improvement in immunologically “cold” tumor microenvironments (37, 38), yet systemic TGF−β blockade carries potential safety and tolerability risks. Therefore, simply enhancing the intensity of immune stimulation without achieving sustained temporal immune input remains insufficient to overcome the long-term immunosuppressive state in bone metastases.

In this context, ^125^I brachytherapy provides a temporally unique treatment modality through continuous LDR exposure, which prolongs ICD signaling and optimizes the temporal coordination between radiotherapy and immune activation, thereby circumventing the limitations of conventional fractionated radiotherapy. Unlike the transient pulses induced by EBRT, we demonstrate for the first time that a single local ^125^I treatment induces dose-and time-dependent ICD, reverses immunosuppressive phenotypes, and significantly enhances CD8^+^T cell infiltration. From day 7 to day 21, HMGB1 release and CALR exposure progressively increased (HMGB1 from 1.66-to 1.88-fold, CALR from 1.70-to 1.96-fold). Recent studies indicate that the concept of ICD has expanded from classical DAMP markers to include systemic ICD molecular signatures and gene expression profiles. ICD scoring systems based on PANX1, AIM2, NLRP3, and other genes have been shown to predict immune infiltration and immunotherapy response in TNBC (39, 40). Consistent with this, our transcriptomic analyses reveal that ^125^I-induced ICD is not an instantaneous event but rather a cascade transitioning from early oxidative stress to sustained endoplasmic reticulum stress and unfolded protein response (UPR) activation. We define this process as “temporal immune remodeling,” characterized by the sequential activation of ICD-related genes including *Ripk3*, *Nlrp3*, *Panx1*, and *Calr*. These findings underscore the importance of sustained immunogenic signaling, rather than peak intensity alone, for effective immune priming. Collectively, our results extend current paradigms, demonstrating that LDR ^125^I brachytherapy enables temporal reprogramming of radiotherapy to intrinsically extend the ICD window without directly perturbing immunosuppressive signaling pathways. This temporally resolved immune remodeling represents a complementary and potentially safer strategy to overcome immune tolerance in the bone metastatic microenvironment.

This work further validates the long theorized but elusive hypothesis that localized radiotherapy to a bone metastasis can generate a systemic immune response capable of controlling distant disease, representing a critical translational goal. Although radiotherapy is proposed as an *in situ* vaccine (35),generating robust abscopal responses in bone metastasis remains exceptionally challenging (41).Clinical evidence indicates that abscopal responses to EBRT are extremely rare (42),and even when combined with ICIs, such responses remain sporadic and infrequent (43).This failure is rooted in the uniquely suppressive bone microenvironment, where high levels of TGF−β and abundant myeloid derived suppressor cells severely limit dendritic cell antigen presentation and T cell priming (44, 45).Our study now demonstrates that ¹²^5^I brachytherapy can overcome this barrier. Using a bilateral tumor model, irradiation of a primary bone lesion significantly suppressed the growth of a distant, non irradiated subcutaneous tumor, with the 0.8 mCi group showing the strongest effect. Histology of distant lesions revealed significant CD4^+^and CD8^+^T cell infiltration, indicating that ¹²^5^I treated bone tumors effectively prime T cells that then circulate and home to remote sites. Mechanistically, the sustained immune activation induced by ¹²^5^I circumvents the limitations of conventional radiotherapy. Our temporal transcriptomic profile shows that inflammatory and DAMP signals induced by ¹²^5^I persist to day 21,matching the time window required for systemic immune activation, a critical window that EBRT fails to provide. This study demonstrates that ¹²^5^I brachytherapy, through its induction of sustained immune activation, effectively reverses the immunosuppressive microenvironment of bone metastases and generates a systemic therapeutic effect capable of inhibiting distant disease. These findings provide a mechanistically grounded strategy for the clinical management of multifocal bone metastases.

Addressing the knowledge gap on the optimal sequencing of radiotherapy and immunotherapy, our systematic comparison reveals that ¹²^5^I serves as a potent immune priming modality. Although ICIs have shown substantial activity in several solid tumors (46),clinical responses in bone metastases remain poor due to the immune−desert phenotype and severe T cell exclusion (47, 48).While radio immunotherapy shows synergy in other malignancies, its benefit in breast cancer bone metastases remains limited (49).Current regimens commonly use EBRT followed by short−interval concurrent ICIs, yet this approach fails to overcome immune resistance and suggests that treatment sequence is a critical determinant of efficacy (45).There is active debate over optimal sequencing, as concurrent ICIs risk damage to immune cells from radiation (30),whereas excessively long intervals reduce synergy (50).The regimen beginning with ¹²^5^I (14 day brachytherapy followed by anti−PD−L1 plus nab−paclitaxel) generated superior tumor control and T cell infiltration compared to the reverse sequence. This 14 day interval aligns with the peak of sustained UPR activation and progressive DAMP elevation identified in our study, creating an optimal immune−favorable context for checkpoint blockade. This finding is consistent with recent insights that low−dose irradiation can enhance the efficacy of PD−1/PD−L1 blockade by remodeling the immune microenvironment (51, 52).Collectively, this work establishes a mechanistically informed sequential paradigm in which ¹²^5^I brachytherapy is used to prime the immune microenvironment, thereby enabling precise timing of subsequent checkpoint blockade to overcome the fundamental barrier of inadequate T−cell activation in bone metastases.

Conventional ICD assessment relies on endpoint measurements, which provide only static information. To overcome this, we developed a CALR targeted molecular probe that specifically recognizes surface exposed CALR, thereby addressing the limited specificity of annexin V and the rapid clearance of HMGB1.*In vitro*, the probe produced characteristic ring like membrane localization in treated cells, confirming its specificity for aberrant CALR exposure. *In vivo*, the probe selectively accumulated in ¹²^5^I treated tumors, and the signal intensity correlated closely with immunohistochemistry. This innovation enables non invasive,real time monitoring of ICD. When combined with PET compatible radiolabeling, it permits dynamic tracking of ICD after brachytherapy, identification of the optimal ICI administration window based on CALR signal peaks, and improved treatment response assessment. In future investigations, longitudinal comparisons using CALR-targeted dynamic imaging at alternative intervals (e.g., 7 and 21 days) will be performed to more precisely define the optimal timing for radiotherapy–immunotherapy sequencing in the clinical setting. The real-time monitoring capability of CALR hotspots will facilitate this approach, supporting the development of personalized, temporally optimized treatment strategies.

This study has several limitations. First, our findings are derived from a single immunocompetent syngeneic mouse model (4T1 in BALB/c), which may not fully capture the heterogeneity and immune complexity of human TNBC bone metastases. Future studies using patient-derived xenografts, additional genetically engineered models, or other metastatic systems are warranted to validate generalizability and explore potential synergy with TGF−β pathway inhibitors. Second, although ¹²^5^I treatment correlated with increased CALR and HMGB1 exposure and enhanced CD8^+^ T cell infiltration, direct causality remains unconfirmed. Mechanistic studies employing knockout, knockdown, or neutralization approaches would clarify their essential roles in immune activation and antitumor efficacy. Third, the 21–28 day observation period demonstrated short-term efficacy but did not assess durable immune memory or long-term tumor control; future work should determine whether ¹²^5^I priming induces persistent systemic immunity to prevent recurrence. Finally, translating preclinical dosing, timing, and sequencing to humans requires careful consideration of interspecies differences. Early-phase exploratory clinical trials could assess safety, feasibility, and preliminary efficacy, while providing a platform to test sequential combinations with ICIs or other immunomodulators. Collectively, these considerations outline clear pathways to extend the translational impact of ¹²^5^I brachytherapy while addressing the inherent limitations of the present study.

In conclusion, this study comprehensively addresses the key knowledge gaps hindering radio immunotherapy for bone metastatic TNBC. We provide quantitative evidence that ¹²^5^I LDR brachytherapy induces a sustained ICD program, overcoming the kinetic limitation of EBRT; demonstrate its capacity to elicit potent systemic immunity and abscopal effects; and define an optimized sequential strategy beginning with ¹²^5^I and followed by ICIs. Coupled with a novel molecular imaging tool for dynamic monitoring, these findings establish ¹²^5^I brachytherapy as a key bridge connecting local treatment with systemic immune modulation. The resulting paradigm provides a novel, clinically translatable framework for overcoming immune resistance in this challenging setting, with the potential to establish a new standard of care for patients with poor prognosis.

## 5 Conclusion

In confronting the formidable challenge of treating immunosuppressive bone metastases in triple-negative breast cancer, this study establishes a paradigm shift by leveraging the unique temporal dynamics of ¹²^5^I brachytherapy. We demonstrate that its continuous low-dose-rate irradiation orchestrates a sustained ICD program, effectively dismantling the “cold” tumor microenvironment and igniting a robust, systemic antitumor immune response. This profound reversal of local immunosuppression is not merely a mechanistic insight but forms the foundation for a transformative therapeutic strategy. By defining an optimized “¹²^5^I-first” sequential regimen, we position brachytherapy as an essential immune-priming modality that creates a critical window to potentiate subsequent anti-PD-L1 therapy, thereby overcoming a central mechanism of immune checkpoint inhibitor resistance.

Crucially, to translate this strategy into clinical practice, we bridged a key technological gap by developing a CALR-targeted molecular imaging probe. This innovation enables the real-time visualization of ICD, moving beyond static endpoint analyses and paving the way for dynamically adaptive, patient-specific treatment scheduling.

Together, our findings crystallize a new treatment paradigm wherein localized ¹²^5^I brachytherapy acts as a pivotal bridge between cytoreduction and systemic immunotherapy. This work provides both a compelling scientific rationale and a directly actionable clinical strategy for overcoming immune resistance in TNBC bone metastasis, with broad implications for the treatment of other refractory metastatic cancers.

## Data Availability

The datasets presented in this study can be found in online repositories. The names of the repository/repositories and accession number(s) can be found in the article/**Supplementary Material**.
