# Supplementary material for: Iodine-125 brachytherapy triggers immunogenic cell death and potentiates anti-PD-L1 immunotherapy in bone metastatic triple-negative breast cancer
Source: Front Immunol. 2026 Mar 31;17:1761538. doi: 10.3389/fimmu.2026.1761538 (PMC13076267; doi:10.3389/fimmu.2026.1761538)
Supplement: Supplementary file 1 [file DataSheet1.docx]

Supplementary Material

# Supplementary Figures and Tables

## Supplementary Tables

**Table 1 PCR primer sequences**

| Gene | Forward primer(5’-3’) | Reverse primer (5’-3’) |
| --- | --- | --- |
| HMGB1 | GCCCATTTTGGGTCACATGG | TGCAGGGTGTGTGGACAAAA |
| CALR | CGAGCCTTTCAGCAACA | CAGACTTGACCTGCCAGAG |
| GAPDH | GCACCGTCAAGGCTGAGAAC | TGGTGAAGACGCCAGTGGA |

## Supplementary Figures


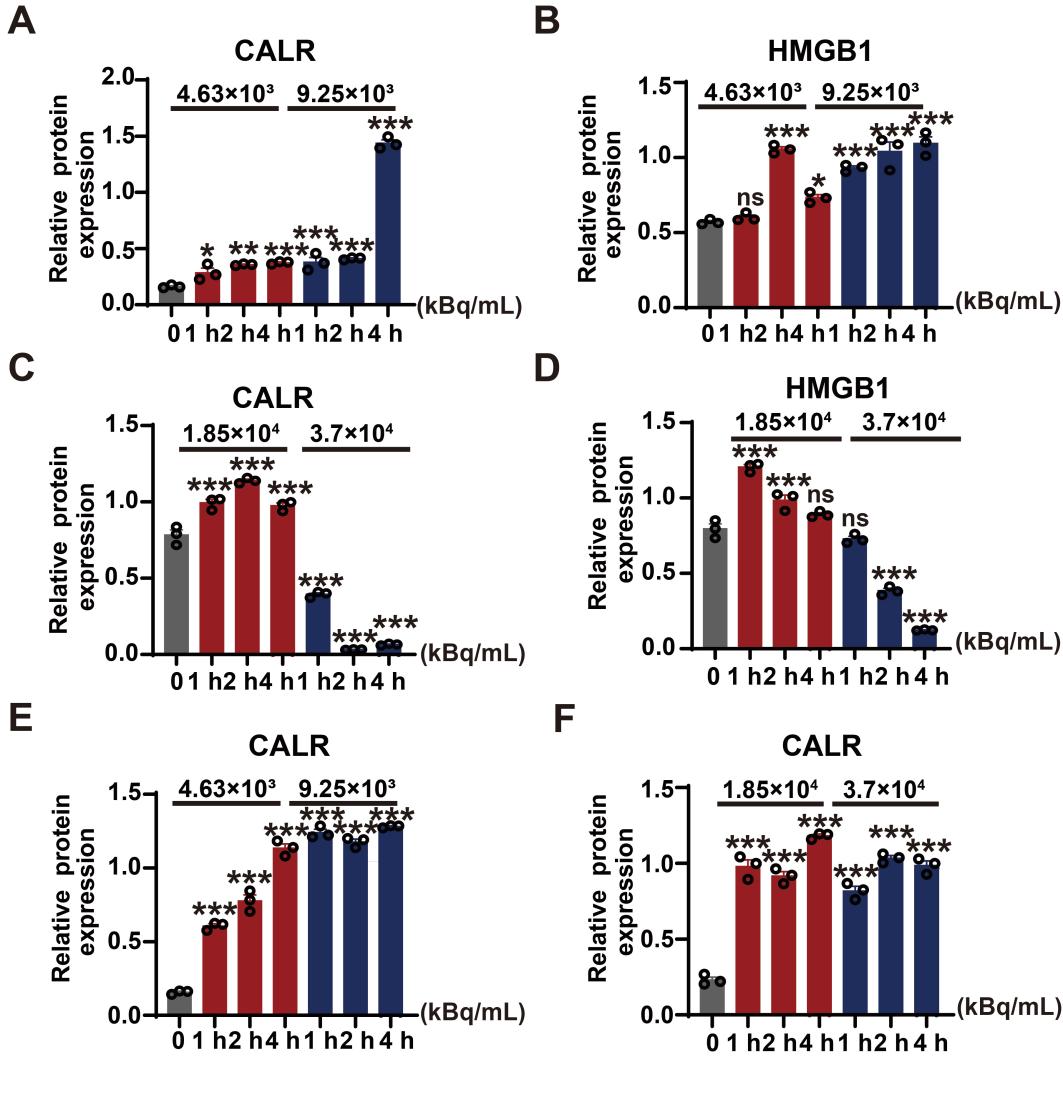


**Supplementary Figure 1**. **^125^I Triggers Immunogenic Cell Death Characterized by Surface CALR Translocation and HMGB1 Secretion.**(A-D) Densitometric quantification of total protein levels of CALR (A, C) and HMGB1 (B, D) determined by Western blot analysis following treatment with ^125^I at low (4.63×10^3^ and 9.25×10^3^ kBq/mL; A, B) or high (1.85×10^4^ and 3.7×10^4^ kBq/mL; C, D) doses at the indicated time points (1, 2, and 4 h).(E-F) Densitometric quantification of membrane-associated CALR levels following low-dose (E) or high-dose (F) ¹²⁵I treatment at the indicated time points.Protein levels were normalized to β-actin for total lysates and to Flotillin-2 for membrane fractions. Data are presented as mean±SEM from three independent experiments. Statistical significance was assessed using one-way ANOVA followed by multiple-comparison correction controlling the false discovery rate (FDR, Benjamini–Hochberg method). ns, not significant; **P*<0.05; ***P*<0.01; ****P*<0.001.


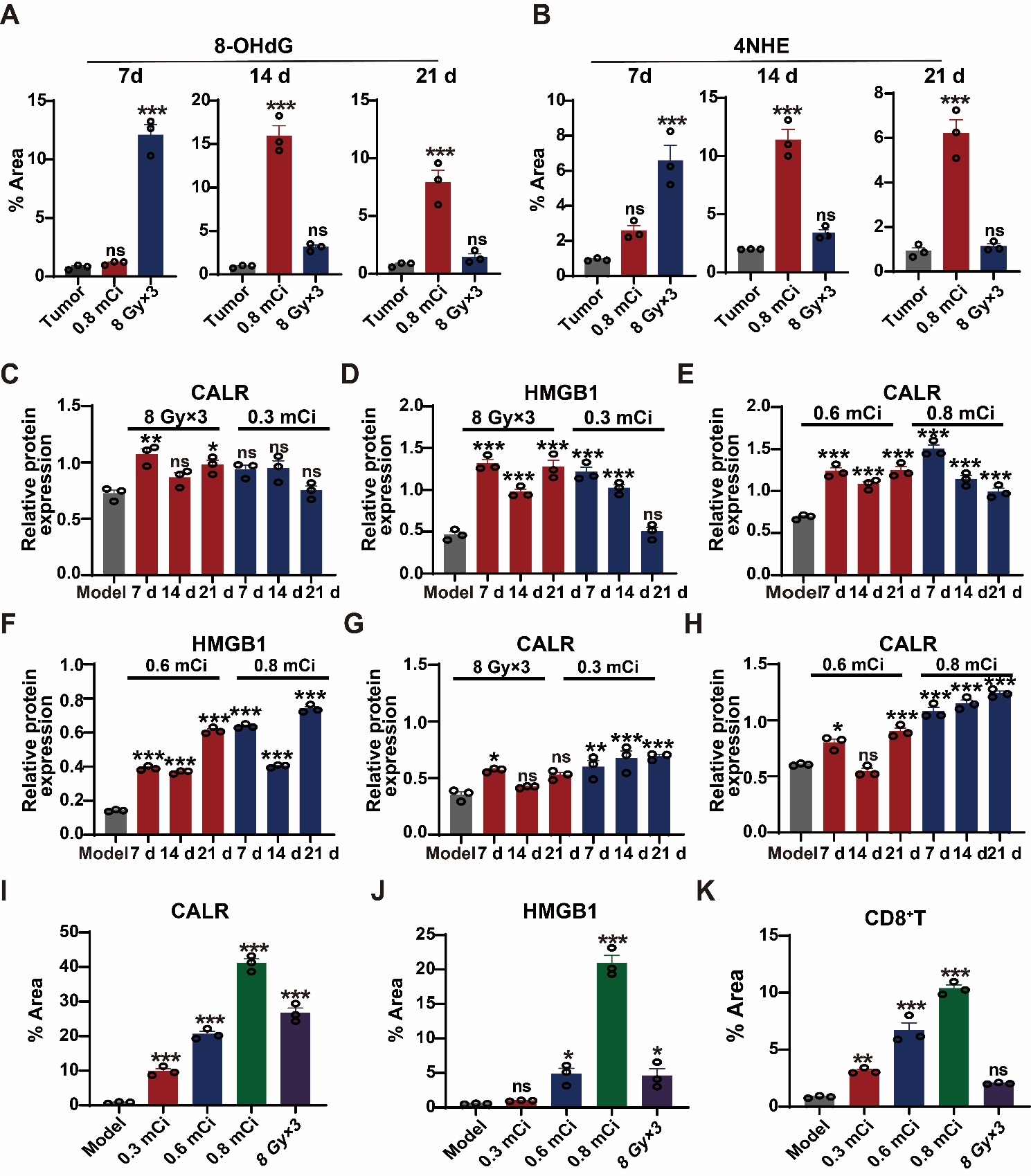


**Supplementary Figure 2**. **^125^I remodels the immunosuppressive tumor microenvironment and promotes robust intratumoral CD8⁺T cell infiltration.**(A-B) Quantitative analysis of oxidative stress markers 8-hydroxy-2′-deoxyguanosine (8-OHdG; A) and 4-hydroxynonenal (4-HNE; B) in tumor tissues by immunohistochemistry at days 7, 14, and 21 following ¹²⁵I brachytherapy or external beam radiotherapy (8 Gy×3).(C-F) Densitometric quantification of total CALR (C, E) and HMGB1 (D, F) protein levels in tumor tissues determined by Western blot analysis following EBRT (8 Gy×3) or local ¹²⁵I implantation at the indicated activities and time points (days 7, 14, and 21). Protein expression was normalized to β-actin.(G-H) Densitometric quantification of membrane-associated CALR protein levels following EBRT or ¹²⁵I brachytherapy , with normalization to Flotillin-2.(I-K) Quantitative analysis of immunohistochemical staining for CALR (I), HMGB1 (J), and CD8⁺ T cells (K) in tumor tissues. For each mouse, values represent the average of three randomly selected non-overlapping fields of view.Data are presented as mean±SEM (n=3 biologically independent mice per group per time point). Statistical significance was assessed using one-way ANOVA followed by multiple-comparison correction controlling the false discovery rate (FDR, Benjamini–Hochberg method). ns, not significant; **P*<0.05; ***P*<0.01; ****P*<0.001.
